# Mechanistic modeling of the bioconcentration of (super)hydrophobic compounds in *Hyalella azteca*

**DOI:** 10.1007/s11356-023-25827-7

**Published:** 2023-02-15

**Authors:** Andrea Ebert, Juliane Ackermann, Kai-Uwe Goss

**Affiliations:** 1 Analytical Environmental Chemistry, Helmholtz Centre for Environmental Research—UFZ, 04318 Leipzig, Germany; 2 Section IV 2.3 “Chemicals”, Umweltbundesamt, 06844 Dessau-Roßlau, Germany; 3 Institute of Chemistry, Martin Luther University Halle-Wittenberg, 06120 Halle, Germany

**Keywords:** *Hyalella azteca*, Bioconcentration factor, 3R principles, Mechanistic modeling, Superhydrophobic compounds, Facilitated transport, Uptake and depuration rate constants

## Abstract

Bioconcentration tests using the freshwater amphipod *Hyalella azteca* as an alternative to conventional fish tests have recently received much attention. An appropriate computational model of *H. azteca* could help in understanding the mechanisms behind bioconcentration, in comparison to the fish as test organism. We here present the first mechanistic model for *H. azteca* that considers the single diffusive processes in the gills and gut. The model matches with the experimental data from the literature quite well when appropriate physiological information is used. The implementation of facilitated transport was essential for modeling. Application of the model for superhydrophobic compounds revealed binding to organic matter and the resulting decrease in bioavailable fraction as the main reason for the observed counterintuitive decrease in uptake rate constants with increasing octanol/water partition coefficient. Furthermore, estimations of the time needed to reach steady state indicated that durations of more than a month could be needed for compounds with a log *K*_ow_ > 8, limiting the experimental applicability of the test. In those cases, model-based bioconcentration predictions could be a preferable approach, which could be combined with in vitro biotransformation measurements. However, our sensitivity analysis showed that the uncertainty in determining the octanol/water partition coefficients is a strong source of error for superhydrophobic compounds.

**Supplementary Information:**

The online version contains supplementary material available at 10.1007/s11356-023-25827-7.

## Introduction

The aim of the 3R principle is to completely avoid animal experiments (replacement), to limit the number of animals (reduction) and their suffering (refinement) in experiments to the absolute minimum (de Wolf et al. 2007). In this sense, it is desirable to replace regulatory fish bioconcentration (or biomagnification) tests with alternative methods. Strong correlations between bioconcentration factors (BCF) measured in fish and in the freshwater amphipod *Hyalella azteca* (Schlechtriem et al. 2019) and good reproducibility (Schlechtriem et al. 2021) make this “model species” in the field of ecotoxicology (Christie et al. 2018) a promising alternative test organism for bioconcentration studies. Only a few milligrams in weight, the organism needs much smaller experimental setups as compared to regular fish tests, and faster uptake and elimination rate constants promise shorter exposure times, making these tests less cost-intensive (Schlechtriem et al. 2019).

In order to be able to plan experiments more efficiently, to check experimental values for plausibility, or to replace animal experiments altogether, it is advantageous to have models that can simulate uptake (*k*_1_) and elimination (*k*_2_) and thus also the bioconcentration factor (BCF) and the time till steady state. A reliable model is needed especially for superhydrophobic compounds, which are difficult to measure experimentally due to their low water solubility, analytical difficulties, and very slow elimination kinetics. Effects such as binding to organic material (total organic carbon (TOC)) within the culture medium, which can extremely reduce the available free aqueous concentration of superhydrophobic compounds (Burkhard 2000; Böhm et al. 2016); possible facilitated transport in the blood or gut (Westergaard and Dietschy 1976; Larisch and Goss 2018); or elimination via feces, which are irrelevant for chemicals with lower octanol/water partition coefficients (*K*_ow_), must be taken into account in the highly hydrophobic range. Although there are models for invertebrates (Arnot and Gobas 2004) or amphipods (Chen and Kuo 2018) in general, we found only one empirical predictive model for *H. azteca* specifically (Lee et al. 2002), which is limited to just a few data points in a narrow range of hydrophobicity, expressed by the *K*_ow_. This empirical model does not allow any meaningful extrapolation beyond its fit range, especially not in the range of highly hydrophobic substances. The model fitted for amphipods in general (Chen and Kuo 2018) has a much broader log *K*_ow_ range from 3.3 to 7.62 but only included 2 datapoints for *H. azteca* in its fit. It is thus unclear how well it will perform for *H. azteca* specifically.

The aim of this work therefore was a mechanistic and not an empirical model of the uptake and elimination rates, as well as the BCF in *H. azteca*. For this purpose, a detailed literature search of the relevant physiological parameters in *H. azteca* was carried out. In order to determine the uptake via the gills or gut, the individual relevant diffusion steps were modeled. The sensitivity of the model to single input parameters was analyzed. The modeling results were then compared with the collected experimental data from the literature measured in *H. azteca* and other existing prediction methods.

## Materials and methods

### Physiological data

A profound literature search was undertaken to collect the necessary physiological data on *H. azteca* to allow for physiologically based modeling of the uptake rate constant, *k*_1_; the whole-body elimination rate constant, *k*_2_; and BCF. In some cases, data have been extrapolated from other amphipods, or in the absence of sufficient data, some data were adopted from fish. To give an overview, data on *H. azteca* and fish are listed side by side in a tabular form in Table 1. If not indicated otherwise, these input parameters were used for the model. References and comments on the data can be found in the Supporting Information, Table S1 and S2.

**Table 1:** Used physiological data in *Hyalella azteca* and fish for modeling

	*Hyalella azteca*	Fish
Body composition		
Wet weight	3 mg	2.2 g
Dry weight	0.8 mg	
Body length	4.5 mm	
Lipid content in organism *C*_*L,*org_	0.02 kg_lipid_/kg_org_	0.04 kg_lipid_/kg_org_
Non-lipid organic matter content in organism *C*_NLOM,org_	0.25 kg_NLOM_/kg_org_	0.16 kg_NLOM_/kg_org_
Water content in organism *C*_*W,*org_	0.73 kg_w_/kg_org_	0.80 kg_w_/kg_org_
Respiration		
Respiration rate	1 mg O_2_/g_wetweight_/h	
* C*_*OX*_	8 mg O_2_/L_w_	11 mg O_2_/L_w_
Temperature	23 °C	13 °C
Ventilation rate constant	3 × 10^4^ L_w_/kg_org_/d	2 × 10^3^ L_w_/kg_org_/d
Food		
Feeding rate of organism *G*_*d*_	0.65 kg_diet,wet_/d/kg_org_	0.01 kg_diet,wet_/d/kg_org_
Dietary assimilation rate of lipids ${\varepsilon }_{L}$	75%	92%
Dietary assimilation rate of NLOM ${\varepsilon }_{N}$	75%	60%
Dietary assimilation rate of water ${\varepsilon }_{W}$	25%	25%
Blood		
Albumin-like protein concentration	41.2 g/L_plasma_	41.2 g/L_plasma_
Total plasma flow ${k}_{\mathrm{bf},\mathrm{tot}}$	252 L_plasma_/kg/d	64 L_plasma_/kg/d
Proportion of total cardiac output,${r}_{\mathrm{bf},\mathrm{gills}}$	1	1
Proportion of total cardiac output,${r}_{\mathrm{bf},\mathrm{gut}}$	0.178	0.178
Organ surface areas		
*A*_gills_	0.05 cm^2^	7 cm^2^
*A*_gut_	0.07 cm^2^	3.8 cm^2^
*A*_skin_	0.14 cm^2^	17 cm^2^

### Model

**Fig. 1:**
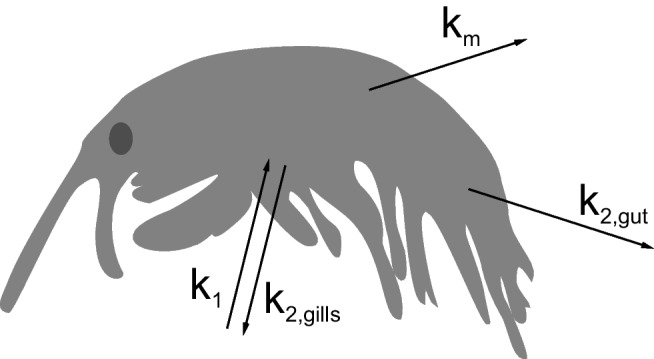
Scheme of dominant uptake and elimination paths in *H. azteca* considered in the developed one-compartment model

We used a one-compartment model for *H. azteca* just as it is typically done for fish in the context of bioconcentration testing. Figure 1 shows the uptake and elimination paths we considered relevant for *H. azteca* for classic BCF measurements: chemical uptake and elimination via ventilation of the gills, elimination via feces, and elimination via biotransformation.

Uptake via diet was not considered, because the animals are fed with uncontaminated diet in BCF studies. We deemed the uptake via the skin irrelevant, since estimated total body area was comparable to the gill area, while a much thicker unstirred water layer as well as several cell layers as compared to the cell monolayer in the gills should lead to a much lower permeation. The general model equation for aquatic organisms (Arnot and Gobas 2004) can thus be simplified to

1 $$\frac{d{c}_{\mathrm{org}}}{dt}={k}_{1}*{f}_{\mathrm{unbound}}*{c}_{w}-\left({k}_{2,\mathrm{gills}}+{k}_{2,\mathrm{gut}}+{k}_{m}\right)*{c}_{\mathrm{org}}$$

where *c*_org_ is the chemical concentration within the organism in kg_chemical_/kg_org_; *c*_*w*_ is the total chemical concentration in water in kg_chemical_/L_w_; *f*_unbound_ is the bioavailable, freely dissolved fraction in water; *k*_1_ and *k*_2,gills_ are the uptake and elimination rate constant via the gills respectively in L_w_/day/kg_org_ and in 1/day; *k*_2,gut_ is the elimination rate constant via feces; and *k*_*m*_ is the elimination rate constant via metabolism. Hydrophobic compounds may bind to organic matter: particulate organic carbon (POC) or dissolved organic carbon (DOC) in water. Typical DOC values in drinking water are about 1 mg DOC/L_w_, and the OECD Guideline 305 allows for a maximum total organic carbon content (TOC = POC + DOC) of 2 mg/L_w_ in the dilution water and 10 mg/L_w_ in the final test medium (OECD 2012). The stronger the chemical binds to TOC, and the higher the TOC content, the lower the actual bioavailable chemical fraction in water. This unbound fraction *f*_unbound_ can be estimated according to (Burkhard 2000)

2 $${f}_{\mathrm{unbound}}={1/(1+C}_{\mathrm{POC}}*0.35*{K}_{\mathrm{ow}}+{C}_{\mathrm{DOC}}*{0.08*K}_{\mathrm{ow}})$$

where *C*_DOC_ is the concentration of DOC in water in kg DOC/L_w_ and *C*_POC_ is the concentration of POC in water in kg POC/L_w_. For the specific experiments with *H. azteca* that we investigate here, we will calculate with a typical DOC concentration of about 1 mg DOC/L_w_ (personal correspondence with Prof. Schlechtriem, flow-through system, Regan et al. (2017) reports more than 90% of TOC in water sources to be DOC). This leads to a substantial decrease in *f*_unbound_ for high log *K*_ow_, see Figure S1.

**Fig. 2:**
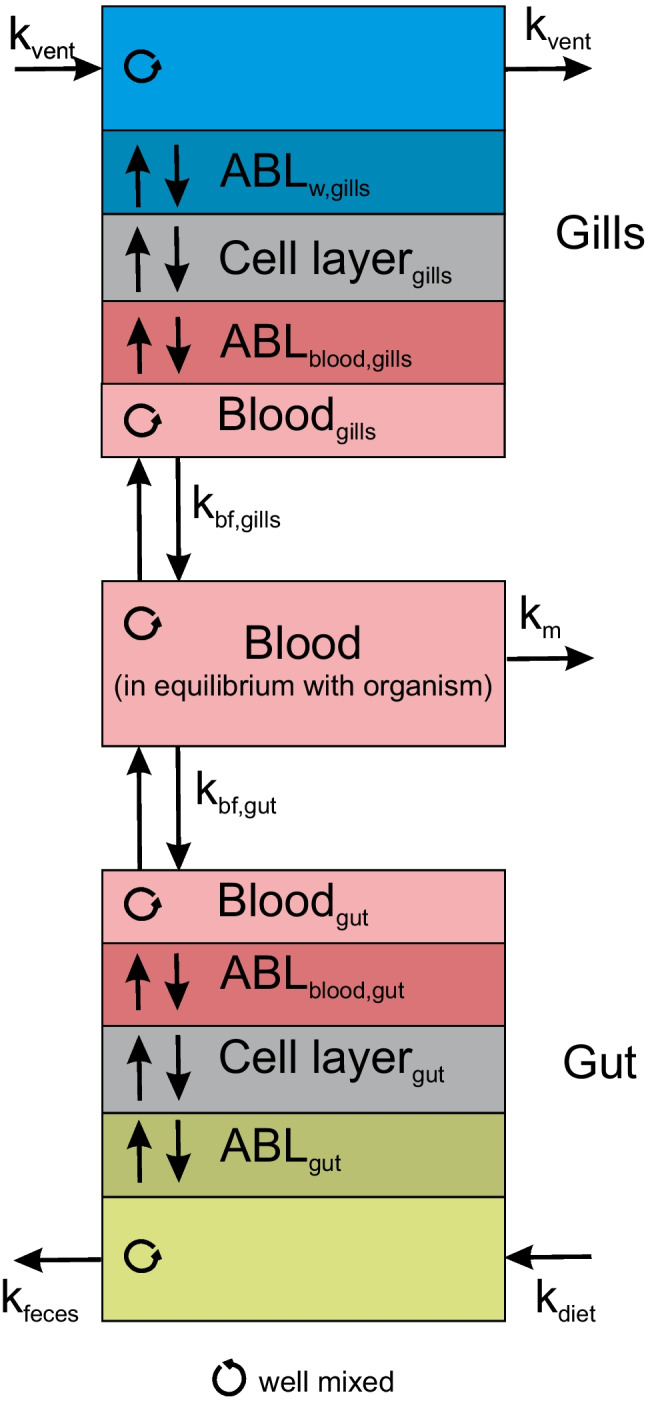
Mechanistic scheme of the processes considered for the derivation of the rate constants for uptake and elimination. Ventilation volume, gut volume, and blood pool are assumed well mixed for the calculations

To derive the rate constants for uptake and elimination in the gills, we mechanistically consider all diffusion resistances between ventilated water and blood connected in series: the unstirred water layer (or aqueous boundary layer, ABL) in water adjacent to the gills, the gill cell monolayer, and the ABL in the blood, see on top of Fig. 2.

The overall uptake rate constant can thus be calculated by inverting the sum of all inversed rate constants:

3 $${k}_{1}=\frac{1}{\frac{1}{{k}_{\mathrm{vent}}}+\frac{1}{{k}_{\mathrm{ABL},w,\mathrm{gills}}}+\frac{1}{{k}_{\mathrm{cell},\mathrm{gills}}}+\frac{1}{{k}_{\mathrm{ABL},\mathrm{blood},\mathrm{gills}}}+\frac{1}{{k}_{\mathrm{bf},\mathrm{gills}}}}$$

where *k*_vent_ is the ventilation rate constant, *k*_ABL,*w*,gills_, *k*_cell,gills_, and *k*_ABL,blood,gills_ are the rate constants for the diffusion through the unstirred water layer in water, through the cell monolayer, and through the unstirred water layer in blood respectively, and *k*_bf,gills_ is the rate constant for blood flow in the gills.

As the diffusive resistances are the same in both directions, *k*_2,gills_ equals *k*_1_ but needs to be divided by *K*_org/w_ to change the reference phase from water to the organism:

4 $${k}_{2,\mathrm{gills}}=\frac{1}{\frac{1}{{k}_{\mathrm{vent}}}+\frac{1}{{k}_{\mathrm{ABL},w,\mathrm{gills}}}+\frac{1}{{k}_{\mathrm{cell},\mathrm{gills}}}+\frac{1}{{k}_{\mathrm{ABL},\mathrm{blood},\mathrm{gills}}}+\frac{1}{{k}_{\mathrm{bf},\mathrm{gills}}}}/{K}_{\mathrm{org}/\mathrm{w}}$$

with *K*_org/w_ being the partition coefficient between the organism and water, calculated according to Arnot and Gobas (2004).

The calculation is quite similar for the elimination via the gut (*k*_2,gut_), with resistances in series being the egestion (rate constant *k*_feces_), the ABL in the gut, the cell monolayer in the gut, the ABL in blood, and blood flow, see Fig. 2 on the bottom.

Again, the reference phase is the organism:

5 $${k}_{2,\mathrm{gut}}=\frac{1}{\frac{1}{{k}_{\mathrm{feces}}}+\frac{1}{{k}_{\mathrm{ABL},w,\mathrm{gut}}}+\frac{1}{{k}_{\mathrm{cell},\mathrm{gut}}}+\frac{1}{{k}_{\mathrm{ABL},\mathrm{blood},\mathrm{gut}}}+\frac{1}{{k}_{\mathrm{bf},\mathrm{gut}}}}/{K}_{\mathrm{org}/\mathrm{w}}$$

where *k*_ABL,*w*,gut_, *k*_cell,gut_, and *k*_ABL,blood,gut_ are the rate constants for the diffusion through the unstirred water layer in water, through the cell monolayer, and through the unstirred water layer in blood, respectively, and *k*_bf,gut_ is the rate constant for blood flow in the gut. Fecal egestion rate *k*_feces_ (kg_feces_/kg_org_/d) was calculated from the feeding rate, assimilation efficiencies and dietary composition as described by Arnot and Gobas (2004). Considering a possible metabolization of the compound inside the organism, the overall elimination rate constant *k*_2_ is modeled as the sum of the single rate constants:

6 $${k}_{2}={k}_{2,\mathrm{gills}}+{k}_{2,\mathrm{gut}}+{k}_{m}$$

where *k*_*m*_ is the whole-body metabolic rate constant.

In steady state, i.e., there is no concentration change with time, Eq. (1) can be set to 0 and rearranged as follows:

7 $$\mathrm{BCF}=\frac{{c}_{\mathrm{org}}}{{c}_{w}}=\frac{{k}_{1}\times {f}_{\mathrm{unbound}}}{{k}_{2,\mathrm{gills}}+{k}_{2,\mathrm{gut}}+{k}_{m}}={\mathrm{BCF}}_{0}\times {f}_{\mathrm{unbound}}$$

The resulting BCF will thus be reduced by the factor of *f*_unbound_ as compared to a BCF that refers to the freely dissolved bioavailable fraction in water, here, marked as BCF_0_. All BCFs were normalized to 5% lipid content multiplying with 0.05 divided by the lipid content of the organism.

In the following, we will go into more detail about the individual diffusion processes.

#### Unstirred water layer

In a well-mixed compartment, the concentration of a solute can be assumed as uniform, yet there will always be an ABL adjacent to the membrane barrier where solute transport is solely governed by diffusive processes (see Section S1, Table S3 and S4 for more information on diffusion). ABL thickness can be lowered by increased agitation or flow (in case of *H. azteca* for example by an increased beating of the pleopods, where the gills reside, and by an increased swimming velocity), but it can never be completely eliminated. Depending on the solute permeability in the membrane, ABL permeability might be a limiting process, which is even more likely for superhydrophobic compounds, because they are expected to have high membrane permeabilities. The rate at which the solute moves across the ABL depends on the diffusion coefficient (*D*), the thickness of the ABL (*d*_ABL_), the diffusion area (*A*), and the concentration difference. The rate constant for diffusion across the ABL in water ${k}_{\mathrm{ABL},w}$ (L_w_/d/kg_org_) can be expressed as follows:

8 $${k}_{\mathrm{ABL},w}=\frac{D*A}{{d}_{\mathrm{ABL}}}/{M}_{\mathrm{org}}$$

where *M*_org_ is the wet weight of the organism.

For the ABL in blood, we additionally assume a facilitation factor (FAC_ABL_) due to the transport of chemical across the ABL bound to albumin-like proteins, see next section for more details.

9 $${k}_{\mathrm{ABL},\mathrm{blood}}={\mathrm{FAC}}_{\mathrm{ABL}}*\frac{D*A}{{d}_{\mathrm{ABL}}}/{M}_{\mathrm{org}}$$

#### Facilitated transport

For very hydrophobic compounds, the highest resistance for cell permeation is not the membrane itself, but the adjacent layers of unstirred water that can only be traversed by passive diffusion. So-called facilitated transport or enhanced diffusion can increase the diffusion across these layers. In parallel to the diffusion of the free chemical, the chemical bound to a carrier is transported by diffusion of the carrier. The resulting facilitation factor (FAC) depends on the partitioning of the chemical between water and carrier and on the diffusion constant of the carrier. If sorption and desorption kinetics between the solute and the carrier are rate limiting, FAC also depends on the ABL thickness, because a thinner ABL corresponds to a shorter residence time.

In the extreme case of extremely slow de-/sorption kinetics as compared to other relevant processes, the fraction bound is not bioavailable at all. This is assumed here in case of TOC, where we expect no facilitated transport.

In the case of de-/sorption kinetics that are not limiting, the FAC_ABL_ can be expressed as follows (Larisch and Goss 2018):

10 $${\mathrm{FAC}}_{\mathrm{ABL}}=\frac{P_{\mathrm{passive}\;\mathrm{diffusion}}^{\mathrm{ABL}}+P_{\mathrm{carrier}\;\mathrm{bound}}^{\mathrm{ABL}}}{P_{\mathrm{passive}\;\mathrm{diffusion}}^{\mathrm{ABL}}}$$

where ${P}_{\mathrm{passive diffusion}}^{\mathrm{ABL}}$ is the permeability across the ABL with no facilitated transport and ${P}_{\mathrm{carrier bound}}^{\mathrm{ABL}}$ is the permeability across the ABL of the chemical bound to the carrier.

11 $$P_{\mathrm{carrier}\;\mathrm{bound}}^{\mathrm{ABL}}=\frac{D_{\mathrm{carrier}}\ast K_{\mathrm{carrier}/\mathrm{water}}\ast c_{\mathrm{carrier}}}{d_{\mathrm{ABL}}}$$

with ${D}_{\mathrm{carrier}}$ as the diffusion coefficient of the carrier in water, ${K}_{\mathrm{carrier}/\mathrm{water}}$ as the carrier/water partition coefficient, ${c}_{\mathrm{carrier}}$ as the carrier concentration, and ${d}_{\mathrm{ABL}}$ as the thickness of the ABL. In case of finite de-/sorption rates, it is necessary to calculate the de-/sorption processes in parallel to the diffusion process. For a detailed calculation of FAC_ABL_ in the ABL in blood see Section S2, Figure S2 and Table S5.

The ABL in the human gut is reported to be 50–2000 µm thick (Kelly et al. 2004). We will assume an ABL thickness of 137 µm in our calculations, because this size was used in determining an empirical correlation to assess the facilitated transport of compounds carried by bile acids in the gut (Westergaard and Dietschy 1976). Details on their prediction can be found in Section S3 and Figure S3.

All resulting facilitation factors are listed in Table S4 and S6.

#### Cell permeation

For the permeation through a cell layer, two parallel diffusion paths are considered: the chemical might either traverse the cell membrane, diffuse through the cytosol, and then traverse the opposite cell membrane, or it might diffuse directly within the membrane without entering the cytosol, the so-called lateral transport (Bittermann and Goss 2017). For less hydrophobic chemicals, it is not energetically favorable to reside in the membrane, the dominating transport path will thus lead through the cytosol. Yet, the membrane itself should not pose a barrier to superhydrophobic compounds, for which lateral transport will therefore dominate.

12 $${P}_{\mathrm{cell}}^{\mathrm{total}}={P}_{\mathrm{cyt}}^{\mathrm{tot}}+{P}_{\mathrm{lateral}}=\frac{1}{{R}_{\mathrm{mem}}/24+{R}_{\mathrm{cyt}}+{R}_{\mathrm{mem}}}+\frac{1}{{R}_{\mathrm{lateral}}}$$

where ${P}_{\mathrm{cell}}^{\mathrm{total}}$ is the total cell permeability, ${P}_{\mathrm{cyt}}^{\mathrm{tot}}$ is the total permeability across the cytosolic route, ${R}_{\mathrm{mem}}$ is the resistances for membrane permeation (the factor 24 accounts for microvilli on the apical membrane), ${R}_{\mathrm{cyt}}$ is the resistance for the diffusion across the cytosol, and ${R}_{\mathrm{lateral}}$ is the resistance across the lateral route. Details can be found in Section S4.

The cell permeation rate constant ${k}_{\mathrm{cell}}$(in L_w_/day/kg_org_) can be expressed as follows:

13 $${k}_{\mathrm{cell}}={P}_{\mathrm{cell}}^{\mathrm{total}}*A/{M}_{\mathrm{org}}$$

where *A* is the surface area of the respective organ and *M*_org_ is the mass of the organism.

#### Ventilation

The ventilation rate constant was calculated from experimentally determined respiration rates from the literature (Johnke 1973; Everitt et al. 2020). We assumed an extraction efficiency for oxygen by the gills of 10%, as a few to 10% efficiency are typical for filter feeders, non-filter-feeding burrow-dwelling invertebrates, and some crustaceans (Barker Jørgensen et al. 1986). If not stated otherwise, we used a temperature of 23 °C and an oxygen concentration *C*_*OX*_ of 8 mg/L for the calculations.

The ventilation rate constant *k*_vent_ was then calculated as follows:

14 $${k}_{\mathrm{vent}}=\frac{\mathrm{Respiratory\; rate}}{{C}_{OX}\times {\mathrm{O}}_{2}\mathrm{extraction \;efficiency}}/{M}_{\mathrm{org}}$$

#### Blood flow

In contrast to the closed circulatory system in fish, crustaceans possess an open system, with nutrients, oxygen, hormones, and cells distributed in the hemolymph (Fredrick and Ravichandran 2012). The classical concept of blood flow through blood vessels like in fish should therefore not be applicable one-to-one. In the absence of any better data for modeling, we will nevertheless as a start calculate the influence of blood flow analogue to fish.

The single rate constants can thereby be expressed by the total plasma flowrate constant *k*_bf,tot_, the percentage of flow through the respective organ *r*_bf,organ_, and the facilitation factor in blood due to the transport of the compound bound to the albumin-like protein:

15 $${k}_{\mathrm{bf},\mathrm{organ}}={k}_{\mathrm{bf},\mathrm{tot}}*{r}_{\mathrm{bf},\mathrm{organ}}/{f}_{\mathrm{unbound}}$$

The factor 1/*f*_unbound_ results from the increased sorption capacity of the blood, as both, compound unbound and bound to the albumin-like protein, are transported at the same rate, and we assume no limitation by de-/sorption kinetics. The total plasma flow is calculated from the organism’s weight and temperature according to Erickson and McKim (1990), see Table S1 for more details. Plasma flow is used instead of total blood flow, because only blood plasma contains albumin, thus de-/sorption reactions to albumin only take place in the blood plasma volume.

### Literature data used for validation

If several exposure concentrations and durations were listed when selecting experimental rate constants and BCF values from the literature, for clarity, we chose to only depict the longest experimental exposure duration (to assure steady state, if possible) and the lowest exposure concentration. The higher exposure concentration values in some experiments had a strong influence on kinetics, BCF, or even mortality due to toxicity. Data extracted from different literature sources regarding the same compound were marked in the respective plots (by a cross). Data from the literature (Landrum and Scavia 1983; Lotufo et al. 2000; Lee et al. 2002; Nuutinen et al. 2003; Schuler et al. 2004; Landrum et al. 2004, 2005; Schlechtriem et al. 2019, 2022; Kosfeld et al. 2020; Johanif et al. 2021) are listed in Table S7, and Figure S4 also depicts the additional concentrations left out in the main plots. Vertical error bars of literature values were taken from the literature as stated; except for azoxystrobin and simazine, these chemicals showed a better fit using a 2-compartment model, and the error represents the difference between the 1- and 2-compartment models. Horizontal error bars depict the uncertainties either for varying experimental values or in their absence, varying predictions between different prediction methods, see Figure S5 for a depiction of the variations in *K*_ow_ predictions for the hydrophobic UV absorbers. Solid-phase microextraction (SPME) techniques are recommended for poorly soluble and highly hydrophobic substances in the OECD guideline 305, yet to our knowledge, none of the literature sources measured the bioavailable fraction of the exposure concentration using SPME; we will thus assume stated *k*_1_ and BCF values to correspond to *f*_unbound_ × *k*_1_ and *f*_unbound_ × BCF_0_, respectively. Only few ionic compounds were measured in *H. azteca*, and key model parameters such as the binding to albumin are not available; the model is therefore restricted to neutral compounds.

For some compounds, measured metabolic rate constants were given. For the remaining compounds, we predicted metabolic rate constants as for fish from the chemical’s SMILES code, using the freely available online platform EAS-E Suite (2022) and the therein implemented QSAR of Brown et al. (2012). These rates might differ between fish and *H. azteca* (Kosfeld et al. 2020), but no prediction tool specific to *H. azteca* was available to assess more reliably a possible importance of metabolism in the overall rate constant.

## Results and discussion

### Prediction of k_1_

#### Comparison of predicted and experimental k_1_

**Fig. 3:**
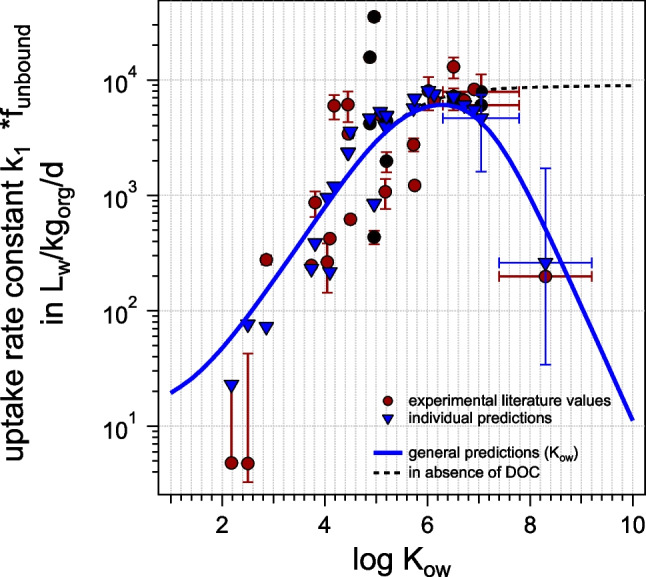
Predicted *k*_1_ according to Eq. (3) for adapted blood flow to account for lesser efficiency of the open circulatory system. *k*_1_ values of same chemicals taken from different literature are marked with a cross

Equation (3) was used to predict the uptake rate constant *k*_1_ in *H. azteca*. In a first approximation, blood flow was calculated analogue to blood flow in fish. This is a very rough estimation, as we would expect the open circulatory system of *H. azteca* to be less effective than the closed system in fish. Indeed, the resulting predicted *k*_1_ seem to be strongly overestimated in the range below log *K*_ow_≈6 (see Figure S6, Table S7 and S8) when comparing them to experimentally determined *k*_1_ from the literature. Exactly in this range, we would expect a limiting transport capacity of the blood (see Larisch et al. (2017) for fish), whereas it should be less limiting for very hydrophobic compounds, because these should strongly bind to the albumin-like protein. It increases the sorption capacity of the blood and thus increases chemical transport via blood flow. If, on the other hand, we reduce blood flow by a factor of 20, which is physiologically plausible due to the possible lower efficiency of the open circulatory system compared to the closed one in fish, the match between experimental and predicted *k*_1_ is much improved (see Fig. 3 and Table S7 and S9). We will therefore use the reduced blood flow as the physiological input in the following modeling, but for completeness, we will show the modeling with blood flow as in fish in the SI. When blood flow was reduced by a factor of 20 as compared to fish, we will in the following refer to it as adapted blood flow. Note that it is not clear whether the less effective transport via the blood flow is only caused by an actual slower blood flow, or if also the binding to the albumin-like protein is reduced as compared to albumin, which would result in a lowered sorption capacity of the blood. In the end, only the low hydrophobic range below log *K*_ow_ 3 is affected by which of these two effects (or which combination) result in a less effective transport via blood flow, see Figure S7. We will assume just an actual decrease in blood flow for the calculations, because this assumption seems to match the two least hydrophobic datapoints best, although the sparsity of data in that range does not allow for a reliable conclusion.

Individual modeling seems to match the experimental data better than the general model trend, which is to be expected and is mainly based on the individually predicted albumin/water binding coefficients.

Many of the outliers are those chemicals which already have bigger uncertainties stated for the experimental *k*_1_ literature value, or which have been taken from different literature sources and strongly deviate from each other. While some of these deviations from one literature value to the other might be explained by different exposure concentrations, we observed an interesting pattern: most *k*_1_ values measured with rather young *H. azteca* at the start of the experiment (younger than 3 weeks) tended to be underestimated by our model, while more mature animals (older than 4 weeks) tended to be well estimated or slightly overestimated (see Figure S8). The younger animals did not yet reach maturity before the start of the experiments, which is reached after about 23–25 days (Othman and Pascoe 2001), and this might cause the discrepancies.

#### Sensitivity analysis of k_1_ prediction

**Fig. 4:**
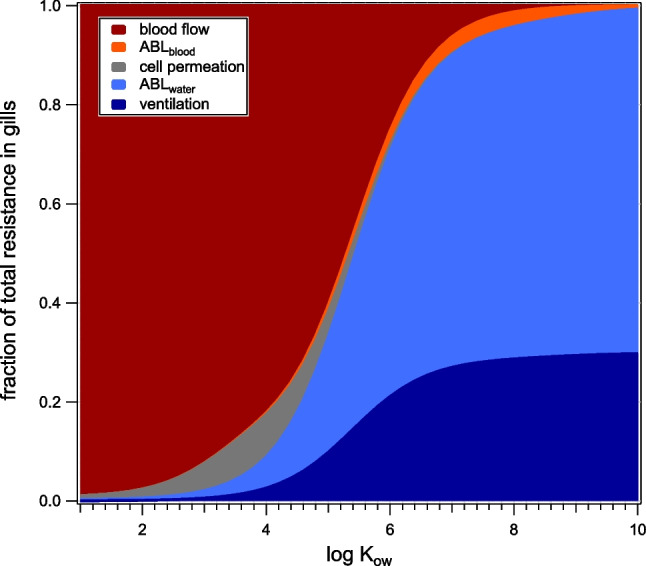
Main resistances for uptake via the gills in *H. azteca* if blood flow is adapted

We also did a sensitivity analysis on other model input parameters, to see the individual impact of parameter uncertainties. Since the model seemed to match to the experimental data better with reduced blood flow, the sensitivity analysis was done both for the initial blood flow modeled as in fish, as well as for the adapted one, see Figures S9 and S10. With the blood flow being the eye of the needle for diffusion of the less hydrophobic compounds, changes in blood flow have the strongest impact in that range, but not for (super-)hydrophobic compounds above log *K*_ow_ 6.5. See Fig. 4 and Figure S11 for a depiction of the main diffusion resistances for the uptake via the gills.

In contrast, in the (super-)hydrophobic range, our model predicts the diffusion through the ABL in water and the ventilation to be the main resistance. For this reason, the model reacts sensitively to changes in the gill area, ABL_water_ thickness, or ventilation rate. Yet, changing these parameters does not seem to improve the overall match, in contrast to the reduced blood flow. Neither changing the ABL thickness nor changing the facilitation factor for facilitated transport by albumin through the ABL have a strong impact, since permeation through the ABL in blood has no significant resistance according to the model. The impact of membrane permeability is also low. Bioaccumulating compounds in the lower *K*_ow_ range that traverse the cell monolayer move directly across the membrane, through the cytosol, and across the opposite membrane. They should be hydrophobic enough to easily traverse the cell membrane but be limited by the diffusion through the cytosol. Compounds that are even more hydrophobic are assumed to take the lateral route, traversing the cell monolayer within the cell membrane, without actually traversing it or the cytosol. If lateral transport is not considered at all, cell permeation becomes the main resistance for the hydrophobic range, which results in a poorer match, see Figure S10f.

#### Comparison to other prediction methods for k_1_

**Fig. 5:**
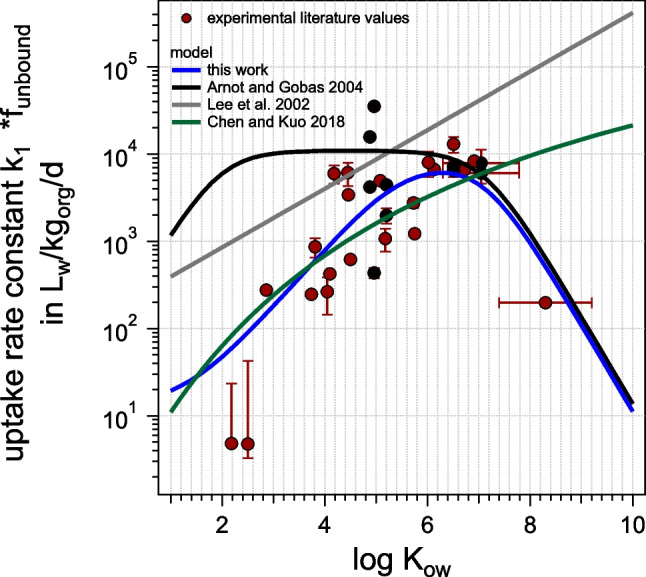
Different models predicting *k*_1_: this work (with adapted blood flow as compared to fish), and models from the literature: Arnot and Gobas (2004), Lee et al. (2002), and Chen and Kuo (2018)

In the literature, other models (see Fig. 5) are available to predict *k*_1_ for *Hyalella azteca*: the model of Arnot and Gobas (2004) can also be applied to invertebrates, Lee et al. made an empirical correlation for *H. azteca* specifically (Lee et al. 2002), and Chen and Kuo (2018) modeled *k*_1_ for amphipods in general. Figure 4 shows the predicted *k*_1_ using these algorithms in comparison with the experimental data: *k*_1_ seem to be strongly overestimated by Arnot and Gobas (2004) in the range below log *K*_ow_ 6. We, therefore, conclude that the general uptake efficiency of the gills for other aquatic animals can thus not simply be applied to *H. azteca*. The correlation by Lee et al. (2002), which was fitted only for a few datapoints, is not able to reproduce the experimental data in the low and high log *K*_ow_ range. In contrast, the general correlation for amphipods (Chen and Kuo 2018) reflects the data surprisingly well. However, different from the prediction method developed in this study, it does not reflect the decrease in *k*_1_ for high log *K*_ow_ chemicals due to a reduced bioavailable fraction in the presence of TOC. In principle, one could adapt the Chen and Kuo method accordingly, see Figure S12.

### Prediction of k_2_

**Fig. 6:**
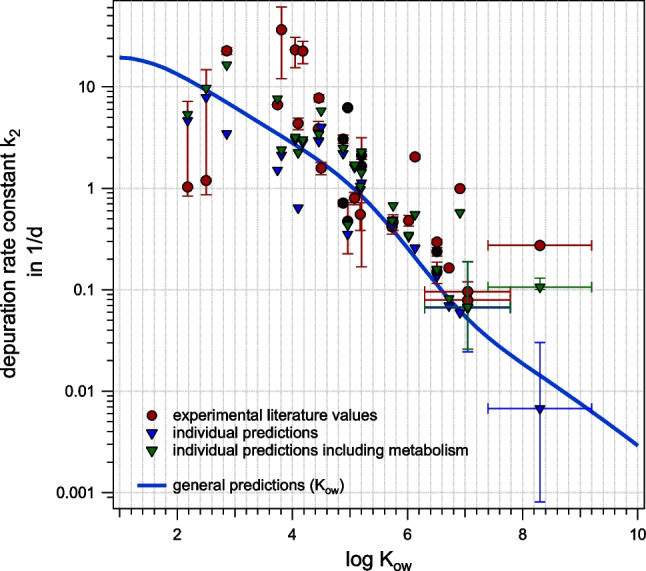
Predicted *k*_2_ according to Eq. (6), in the absence (blue) or presence (green) of metabolism, alongside experimental *k*_2_ (red) for adapted blood flow to account for lesser efficiency of the open circulatory system. Same chemicals taken from different literature are marked with a cross

Equation (6) was used to predict the depuration (elimination) rate constant *k*_2_ in *H. azteca*. In Fig. 6, k_2_ resulting from a general prediction for log *K*_ow_ ranging from 1 to 10 are shown as a trend line, and individual predictions for specific chemicals for which experimental data are available are shown as datapoints. For specific chemicals, it is also possible to derive estimates of metabolism, and by this, metabolism can be considered in the prediction of *k*_2_ for these cases (green datapoints). If blood flow is modeled analogue to fish, *k*_2_ are in general overestimated in the less hydrophobic range (see Figure S13), but then again, the match improves with adapted blood flow (see Fig. 6), which is consistent with what we have seen in *k*_1_. Generally, the modeled *k*_2_ agree quite well with the experimental data, but some data are underestimated (see Table S7 and S9 for detailed values). Most of the underestimated *k*_2_ might be explained by the influence of metabolism. This concerns especially the UV absorbers UV-234 and UV-329, which show much higher *k*_2_ in the experiment than would be expected from elimination via the gills and feces alone. Our predictions underestimate *k*_2_ by a factor of 41 for UV-234 and a factor of 17 for UV-329. Although no in vivo or in vitro data on metabolism were available for these compounds, empirical prediction methods for whole-body metabolic rates in fish suggest relevant metabolism (Brown et al. 2012; EAS-E Suite 2022). Note that these estimated rates can just give a qualitative suggestion; the values per se might differ from fish and also due to different experimental temperatures. Five of in total six available experimental *k*_*m*_ values were underestimated by the empirical correlation for biotransformation. This might also have been the case for other compounds such as fluorene, for which significant metabolic activity was reported (Lee et al. 2002), while the predicted metabolic rate was insignificant. Unfortunately, the experimental data is not sufficient to evaluate the performance of our modeling of the elimination via the gut, *k*_2,gut_. According to our model, elimination via the feces/gut will only exceed elimination via the gills at about log *K*_ow_ 7 (see Figure S14), and only UV-234 would in principle qualify for that evaluation, but its *k*_2_ value is likely dominated by metabolism. It also remains unclear if the same reduction factor of 20 for the blood flow should be used in the gut as for the gills.

### Bioconcentration factor

**Fig. 7:**
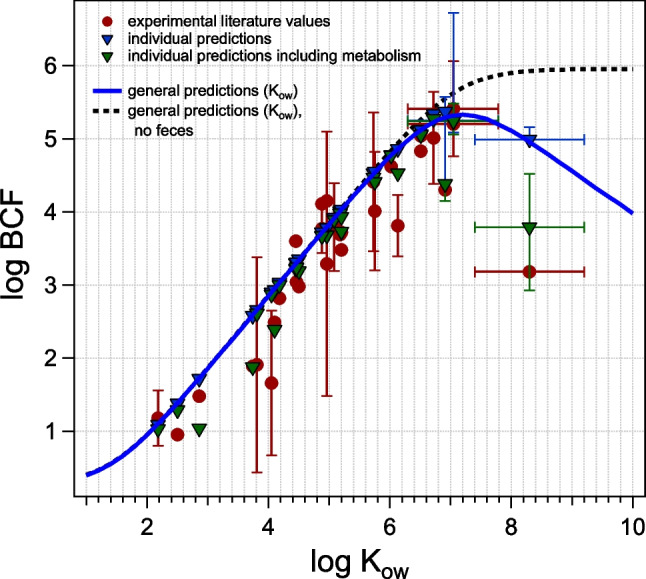
Predicted log BCF according to Eq. (7), in the absence (blue) or presence (green) of metabolism, alongside experimental *k*_2_ (red) for adapted blood flow to account for lesser efficiency of the open circulatory system

The experimental and predicted log BCF are depicted in Fig. 7 and Figure S15. The general trend and individual predictions in the absence of metabolism differ only minimally between an assumed blood flow analogue to fish or the adapted one and only in the highly hydrophobic range where *k*_2_ is dominated by *k*_2,gut_, because the reduced blood flow affects both elimination paths slightly differently. In the less hydrophobic range, where uptake and elimination are dominated by the gills alone, the effect of blood flow (or any other diffusive resistances) cancels out. The uptake and elimination in the low *K*_ow_ range are dominated by the gills, which results in a BCF equal to *K*_org/w_ × *f*_unbound_. The influence of TOC alone would only lead to a plateau (constant BCF at high *K*_ow_), but the combination with feces as an additional elimination path will lead to a decrease in BCF with *K*_ow_ at high *K*_ow_. Taking metabolism into consideration, the adapted blood flow results in a better match between experimental and predicted BCF, since relevant metabolic rates are insignificant in comparison to the much higher predicted *k*_2_ if the blood flow is not assumed to be reduced as compared to fish.

### Comparison to fish

**Fig. 8:**
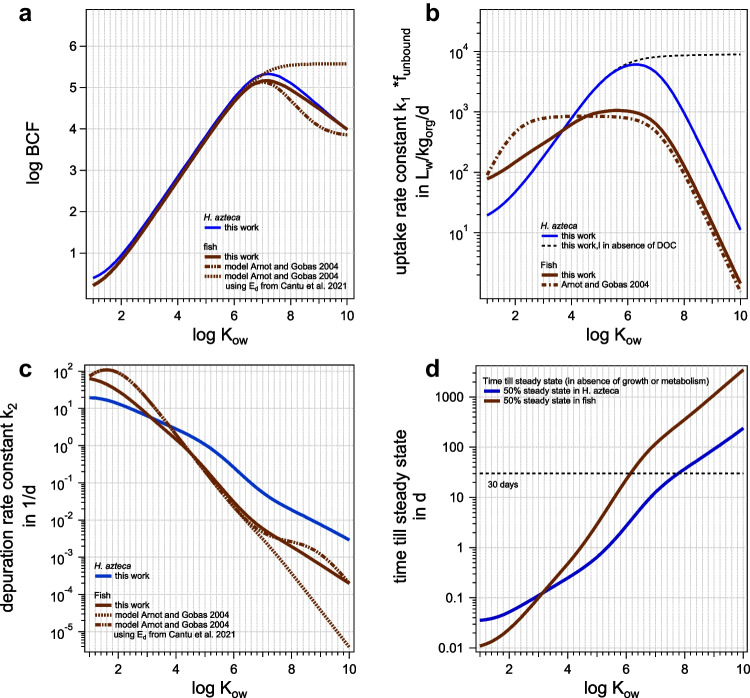
Comparison between *H. azteca* and fish **a** BCF, **b** uptake rate constant *k*_1_, **c** depuration rate constant *k*_2_, **d** time till 50% of steady state is reached. Calculations were done in the presence of 1 mg DOC/L_w_, and fish diet was 1% of organism wet weight per day

Overall, the BCF predicted with our model (in the absence of metabolism) for *H. azteca* and fish are quite similar, see Fig. 8a for a comparison of our model in *H. azteca* to our model in fish, as well as to a model in fish from the literature (Arnot and Gobas 2004). For detailed values see Tables S10 and S11. The differences in the model for fish from Arnot and Gobas (2004) result from different empirical correlation for the dietary uptake efficiency (Arnot and Gobas 2004; Cantu and Gobas 2021) used in the modeling. They determine the influence of feces, and the difference between both models illustrates the strong uncertainties in the superhydrophobic range above log *K*_ow_ 6.5, even for fish. Although predictions thus suggest a good comparability between BCF in *H. azteca* and fish, due to the unclear data basis in this range, especially for *H. azteca*, as discussed above, a conclusive comparison is not possible.

Also experimentally, a good correlation between BCF in *H. azteca* and fish was observed (Schlechtriem et al. 2019). Yet, in the range of compounds with log *K*_ow_ above 4.5, some compounds were determined to be bioaccumulative in *H. azteca*, but not in fish. The authors assumed that the BCF which were higher in *H. azteca* than fish might be explained by a limited biotransformation capacity of amphipods (Schlechtriem et al. 2019). Another factor might be involved regarding the influence of metabolism: since *k*_2_ (not including metabolism) above log *K*_ow_ 4.5 are predicted much higher in *H. azteca* than in fish (up to one order of magnitude (see Fig. 8c), and similar whole-body metabolic rate constants would have less impact in *H. azteca* than in fish.

These higher *k*_2_ (not including metabolism) also have another consequence: steady state for hydrophobic compounds (in the absence of metabolism or growth) is reached faster in *H. azteca* than in fish. Nevertheless, the time to reach 50% of steady state increases with *K*_ow_, and even for *H. azteca*, at log *K*_ow_ greater than 7.8, the time till steady state amounts to more than 30 days. This is impractical for standard experiments, and for extremely high *K*_ow_, the time even exceeds the lifespan of *H. azteca*. For fish, already at log *K*_ow_ of 6.2, time till steady state reaches 30 days, see Fig. 8d.

One further advantage of the increased *k*_2_ is that the growth rate is less significant. Especially in the hydrophobic range, the dilution via growth might exceed other elimination paths, making a correction for growth extremely unreliable. For *H. azteca*, it is thus recommended to use mature animals, which grow much slower than young ones. Analyzing data on growth from Othman and Pascoe (2001), we derived growth rates of 0.003 1/day for old *H. azteca* (fit to day 58–187) and greater than 0.08 1/day for young ones. For *H. azteca*, growth rate for the old amphipods would thus only exceed *k*_2_ at log *K*_ow_ greater than 10. Growth rates of fish of 0.01 1/d, as used in the study for superhydrophobic UV absorbers UV-234 and UV-329, in the absence of metabolism, would, according to our model, already exceed *k*_2_ at log *K*_ow_ 6.6, making a correction for growth extremely unreliable or even impossible in the superhydrophobic range.

## Conclusion

Our model’s prediction of *k*_1_ in *H. azteca* seems to be reliable, as long as a reduced blood flow compared to fish is assumed. We can also recommend the general empirical correlation for amphipods (Chen and Kuo 2018), but one should multiply it by the unbound fraction to account for bioavailability. The strongest uncertainty is for the (super-)hydrophobic range (log *K*_ow_ > 6.5) due to the influence of TOC. A reliable determination of the bioavailable fraction is difficult, since TOC levels and the nature of TOC present in water will differ from experiment to experiment, especially between different laboratories that use different water sources and experimental setups. Even aside from uncertainties in the prediction method for the binding to TOC, the difficulty to predict or measure *K*_ow_ in the superhydrophobic range will lead to strong uncertainties. A SPME measurement of bioavailable fraction is thus strongly recommended for specific experiments.

The prediction of *k*_2_ in the superhydrophobic range is complicated as well. Both growth and feces gain more importance and might even dominate elimination altogether. Some experimentalists try to avoid this problem by not feeding the amphipods during the experiment (Nuutinen et al. 2003), but this is not in the interest of “refinement,” to enhance animal welfare. We recommend to use *H. azteca* older than 2 months in the superhydrophobic range, as was done in the measurement of UV absorbers UV-234 and UV-329 (Schlechtriem et al. 2022), which should lead to insignificant growth or allow for growth correction. Feces as an additional elimination path, without the additional uptake route via a contaminated diet, is unrealistic and might potentially lead to a misclassification of bioaccumulative substances, not only in *H. azteca*, but also in fish. Yet, at least our current modeling suggests that for log *K*_ow_ < 10, the elimination via feces should not be high enough to cause a misclassification, except for extreme cases of nearly 10 mg TOC/L_w_, which should not be reached in a flow-through system. Nevertheless, our model for the elimination via feces could not be verified in that range due to the lack of experimental data measured in *H. azteca*. If more data become available in the future, this problem should be re-evaluated.

BCF seem comparable to fish in the absence of metabolism, yet differences in metabolism and its relative importance as compared to the overall *k*_2_ might lead to tendentially higher BCF than in fish, and *H. azteca* might thus be a more conservative test organism. A strong advantage of the test in *H. azteca* as compared to the BCF test in fish is the shorter time till steady state, which should allow to measure compounds of higher hydrophobicity than in fish. Nevertheless, for extremely high *K*_ow_, experimental tests of several months will reach a limit of feasibility in the absence of metabolism and with insignificant growth. In the extreme case, required experiment duration would exceed the lifespan of the amphipods. In that case, combining predicted *k*_1_ with metabolic rate constants estimated from in vitro depletion rate constants (Trowell et al. 2018; Kosfeld et al. 2020) might be a perspective.

## Supplementary Information

Below is the link to the electronic supplementary material.

Supplementary file1 (PDF 2074 KB)

## Data Availability

The data is publicly available, and all source of data used in this research is given in the manuscript.
