# Supplementary material for: Mechanistic modeling of the bioconcentration of (super)hydrophobic compounds in Hyalella azteca
Source: Environ Sci Pollut Res Int. 2023 Feb 15;30(17):50257–68. doi: 10.1007/s11356-023-25827-7 (PMC10104946; doi:10.1007/s11356-023-25827-7)
Supplement: Supplementary file 1 — Supplementary file1 (PDF 2074 KB) [file 11356_2023_25827_MOESM1_ESM.pdf]

Supporting Information for

Mechanistic modeling of bioconcentration of (super)hydrophobic compounds in *Hyalella azteca*

Andrea Ebert*[,i], Juliane Ackermann[ii], Kai-Uwe Goss[i,iii]

[i] Analytical Environmental Chemistry, Helmholtz Centre for Environmental Research – UFZ, D-04318 Leipzig, Germany
[ii] Section IV 2.3 „Chemicals“, Umweltbundesamt, D-06844 Dessau-Roßlau, Germany
[iii] Institute of Chemistry, Martin Luther University, D-06120 Halle, Germany
* Corresponding author. Analytical Environmental Chemistry, Helmholtz Centre for Environmental Research – UFZ, D-04318 Leipzig, Germany
E-mail: andrea.ebert@ufz.de

Content

List of figures 1

List of tables 2

List of abbreviations 3

S1 Diffusion 9

S2 Facilitation factor in ABL_{blood} 13

S3 Bile acids as carriers in the gut 16

S4 Cell permeation 18

Supporting References 34

List of figures

Figure S1. Fraction unbound (or bioavailable fraction) in water for the different indicated DOC concentrations in water 9

Figure S2 Diffusion steps in gills (left) and facilitated transport of the chemical across the ABL in blood (right) 14

Figure S3 Correlation between calculated log FAC and experimental log Kow 16

Figure S4 Influence of different chemical concentrations in the exposure medium on k1 21

Figure S5 Octanol/water partition coefficients predicted for UV-329 and UV-234 using different prediction tools. 21

Figure S6 Predicted k1 according to Eq. (3) for blood flow as in fish. k1 values of same chemicals taken from different literature are marked with a cross. 22

Figure S7 Differences in modeled k1 if transport by blood flow is reduced due to a reduced binding to albumin and thus a reduction in sorption capacity, or by an actual reduction in blood flow 25

Figure S8 Differences in k1 due to organism age at the start of the experiment. Young, immature amphipods tend to have higher k1 than mature ones. 25

Figure S9 Sensitivity analysis in *H. azteca*, blood flow calculated as in fish. 26

Figure S10. Sensitivity analysis in *H. azteca*, calculated with adapted blood flow. 27

Figure S11. Main resistances for uptake via gills in *H. azteca* if blood flow is calculated as in fish.... 28

Figure S12. Different models predicting k1........ 28

Figure S13. Predicted k2 for blood flow modeled as in fish. 29

Figure S14. Relative importance of elimination paths in *H. azteca*........ 29

Figure S15. Predicted log BCF for blood flow modeled as in fish. 30

List of tables

Table S1. Physiological data in *Hyalella azteca*: references and comments. 5

Table S2. Physiological data in fish: references and comments........ 7

Table S3: SMILES, Molecular weight MW and log K_{ow} of chemicals measured in *H. azteca* 10

Table S4: Diffusion coefficient D_w, albumin/water partition coefficient $K_{albumin/w}$, aqueous solubility S_w, melting temperature Tm, subcooled solubility $S_{subcooled}$, calculated facilitation factors by bile micelles in the gut FAC_{mic} and albumin in blood $FAC_{ABL,albumin}$ for hydrophobic chemicals........ 12

Table S5: System of equations considering de-/sorption kinetics to calculate FAC in blood. 15

Table S6. Calculated facilitation factors by bile micelles in the gut FAC_{mic} and albumin in blood $FAC_{ABL,albumin}$ depending on the octanol/water partition coefficient log K_{ow} 17

Table S7 Chemical name, experimental aqueous exposure concentration c_{exp}, experimental rate constants k1 and k2 and respective errors, experimental whole body metabolic rate constant $k_{m,exp}$, experimental kinetic log BCF and respective error from several literature sources as indicated. 18

Table S8 Chemical name, experimental uptake rate constant k1 considering 1 mg DOC /L_w and elimination rate constant $k_{2,gills}$+$k_{2,gut}$ (considering ventilation and feces) and k2 (considering ventilation, feces and metabolism), log BCF and log BCFm not considering/considering metabolism respectively, predicted metabolic rate constant, predicted metabolic half-life. Blood flow is assumed as in fish. 22

Table S9 Chemical name, experimental uptake rate constant k1 considering 1 mg DOC /L_w and elimination rate constant k2,gills+k2,gut (considering ventilation and feces) and k2 (considering ventilation, feces and metabolism), log BCF and log BCF_m not considering/considering metabolism respectively, predicted metabolic rate constant, predicted metabolic half-life. Blood flow is assumed reduced by a factor of 20 as compared to fish. 23

Table S10: *Hyalella azteca*: Calculated uptake rate constant k1 for TOC content of 1 mg DOC/L_w, depuration rate constant k2, log BCF, and estimated times till 50% of steady state is

reached, depending on the octanol/water partition coefficient log Kow. Calculations were done with adapted blood flow. 30

Table S11 . Fish: Modeled uptake rate constants k1 for TOC content of 1 mg DOC/L_w, the log. BCF, k2 and estimated times till 50 % of steady state is reached, depending on the octanol/water partition coefficient log K_{ow}. 32

List of abbreviations

**ABL**	aqueous boundary layer
**A_{gills}**	gill surface area
**A_{gut}**	gut surface area
**A_{skin}**	total body surface area
**BCF**	bioconcentration factor
**BW**	organism body weight
**$C_{L,org}$**	total lipid content of organism
**$C_{NLOM,org}$**	non lipid organic matter content of organism
**C_{OX}**	oxygen concentration in water
**$C_{W,org}$**	water content of organism
**d**	day
**D**	diffusion coefficient in water
**DDD**	dichlorodiphenyldichloroethane
**DDE**	dichlorodiphenyldichloroethylene
**DDT**	dichlorodiphenyldichloroethylene
**DOC**	dissolved organic matter content in water
**ε_L**	dietary assimilation rate of lipids
**ε_N**	dietary assimilation rate of NLOM
**ε_W**	dietary assimilation rate of water
**FAC**	facilitation factor
**$FAC_{albumin}$**	facilitation factor with albumin in blood as carrier
**FAC_{mic}**	facilitation factor with bile micelles in gut as carriers
$f_{unbound}$	unbound fraction
H. azteca	*Hyalella azteca*

**ABL**	aqueous boundary layer
**k1**	uptake rate constant
**k2**	depuration rate constant
$k_{2,gills}$	elimination rate constant via gills
$k_{2,gut}$	elimination rate constant via gut
$k_{ABL,blood,gills}$	rate constant for the diffusion through the unstirred water layer in blood in the gills
$k_{ABL,blood,gut}$	rate constant for the diffusion through the unstirred water layer in blood in the gut
$k_{ABL,w,gills}$	rate constant for the diffusion through the unstirred water layer in water adjacent to the gills
$k_{ABL,w,gut}$	rate constant for the diffusion through the unstirred water layer in water adjacent to the gut
$K_{alb/w}$	albumin/water partition coefficient
$k_{bf,gills}$	rate constant for the blood flow to/from the gills
$k_{bf,gut}$	rate constant for the blood flow to/from the gut
$k_{cell,gills}$	rate constant for the diffusion through the cell monolayer in the gills
$k_{cell,gut}$	rate constant for the diffusion through the cell monolayer in the gut
k_{diet}	dietary uptake rate constant
k_{feces}	egestion rate constant
$kg_{chemical}$	kilogram chemical
kg_{org}	kilogram organism
k_m	elimination rate constant via metabolism
$K_{org/w}$	organism/water partition coefficient
K_{OW}	octanol/water partition coefficient
k_{vent}	ventilation rate constant
L_w	liter water
M_{org}	wet weight of organism
**MW**	molecular weight
**NLOM**	non lipid organic matter
O_2	oxygen

**ABL**	aqueous boundary layer
**PCB153**	2,2',4,4',5,5'-hexachloro-1,1'-biphenyl
**PCB77**	3,3',4,4'-tetrachlorobiphenyl
**POC**	particulate organic matter content in water
**R**	gas constant
**SMILES**	simplified molecular input line entry specification
**$S_{subcooled}$**	subcooled solubility
**S_w**	aqueous solubility
**t50**	time till 50% of steady state is reached
**t90**	time till 90% of steady state is reached
**T_m**	melting temperature
**TOC**	total organic matter content in water
**UV-234**	2-(2h-benzotriazol-2-yl)-4,6-bis(2-phenyl-2-propanyl)phenol
**UV-329**	2-(2h-benzotriazol-2-yl)-4-(1,1,3,3-tetramethylbutyl)phenol

Table S1. Physiological data in *Hyalella azteca*: references and comments.

	Hyalella azteca used value	references and comments
Body composition		
Wet weight	3 mg	Mean value taken from (Schlechtriem et al., 2019). Animals were between 1 week and over 2 months old, which should correspond to a weight range of about 0.5 -7 mg (Othman & Pascoe, 2001).
Dry weight	0.8 mg	Dry to wet weight ratio in *H. azteca* is 0.27 (Landrum & Scavia, 1983), or about 1:4 (Othman & Pascoe, 2001). Value calculated for 3 mg wet weight.
Body length	4.5 mm	Body length corresponding to 3 mg wet weight (Othman & Pascoe, 2001).
Lipid content in organism $C_{L,org}$	0.02 kg_{lipid}/kg_{org}	Mean lipid content from (Schlechtriem et al., 2019).
Non Lipid Organic Matter content in organism $C_{NLOM,org}$	0.25 kg_{NLOM}/kg_{org}	Calculated as the total content minus lipid and water content.
Water content in organism $C_{W,org}$	0.73 kg_{W}/kg_{org}	Calculated from ratio of dry weight to wet weight.

Respiration		
Respiration rate	1 mg O_2/$g_{wetweight}$/h	1.3 mg O_2/$g_{wetweight}$/h at 23°C (Everitt et al., 2020)<br><br>45uL O_2/$g_{dryweight}$/min (with oxygen density of 1.1 g/L, this corresponds to 0.8 mg O_2/$g_{wetweight}$/h) at 25°C (Johnke, 1973)<br><br>205 mg O_2/$g_{wetweight}$/h (Gauthier et al., 2016) [not considered, outlier]
C_{OX}	8 mg O_2/L_w	Measured oxygen concentration C_{OX} between 6.9 and 9.3 mg/L (Schlechtriem *et al.*, 2019)
temperature	23°C	Whole temperature range 18 - 27°C
Ventilation rate constant	$3*10^4$ L_w/kg_{org}/d	Calculated from respiration rate using Eq. 14.<br><br>Quite similar to $2*10^4$ L_w/kg_{org}/d, calculated using the empirical correlation (Arnot & Gobas, 2004):<br><br>$$k_{vent} = \frac{1400 * BW^{-0.35}}{C_{OX}}$$<br>Where BW is the weight of the organism in kg, and C_{OX} the oxygen concentration in mg O_2/L_w.
Food		
feeding rate of organism G_d	0.65 $kg_{diet,wet}$/d/kg_{org}	Feeding was done ad libitum in (Schlechtriem et al., 2019, 2022), with fish feed embedded in an agar matrix (Kampfraath et al., 2012), consisting of 23 ml water, 1500 mg ground and 500 mg agar-agar (Schlechtriem et al., 2022). We calculated the final composition of the DECOTAB as 0.924 kg_{water}/kg_{diet}, 0.07 kg_{NLOM}/kg_{diet}, and 0.007 kg_{lipid}/kg_{diet}. Assuming a similar feeding rate G_d as for A. aquaticus with 3 $mg_{diet,dry}$/d/(5 animals) (Kampfraath et al., 2012), with a wet weight of 12 mg for A. aquaticus of 8 mm length (Fitzpatrick, 1968), this leads to a feeding rate of 0.05 $kg_{diet,dry}$/d/kg_{org}, or 0.65 $kg_{diet,wet}$/d/kg_{org}. The feeding rate calculated after (Arnot & Gobas, 2004) is quite similar, with resulting 0.59 $kg_{diet,wet}$/d/kg_{org}<br><br>$$G_d = 0.022 * MW^{0.85} * \exp(0.06 * T)$$
dietary assimilation rate of lipids ε_L	75%	From (Arnot & Gobas, 2004) for aquatic invertebrates
dietary assimilation rate of NLOM ε_N	75%	From (Arnot & Gobas, 2004) for aquatic invertebrates
dietary assimilation rate of water ε_W	25%	From (Arnot & Gobas, 2004) for all fresh- water species
Blood		
Albumin-like protein concentration	41.2 g / L_{plasma} [b]	Albumin protein concentration in fish from (Escher et al., 2011), assuming a protein density of 1.39 kg/L, this corresponds to 0.0296 $L_{albumin}$/ L_{plasma}.

		Albumin-like protein concentration assumed the same as albumin in fish. The diffusion coefficient of the albumin-like protein in water is assumed equal to that of albumin 6.3*10^{-7} cm^2/s (Gaigalas et al., 1992).<br>Note that this is a very rough assumption.
Total plasma flow $k_{bf,tot}$	252 L_{plasma}/kg/d	Cardiac output Q_B was calculated as for fish after (Erickson & McKim, 1990): $Q_B = (0.23 * T - 0.78) * (\frac{M_{org}}{500})^{-0.1}$ with M_{org} in g and T in °C.<br>To covert L_{blood} in L_{plasma}, Q_B was multiplied by 0.7 as in fish (Gingerich et al., 1987).
$r_{bf,gills}$	1	As in fish, (Larisch et al., 2017)
$r_{bf,gut}$	0.178	As in fish, (Larisch et al., 2017)
Organ surface areas		
A_{gills}	0.05 cm^2	Empirical correlation developed for the amphipod Gammarus fossarum (SUTCLIFFE, 1984):<br>$$A_{gills} = 5.4223 * BW_{dry}^{0.79}$$<br>Where BW_{dry} is the dry weight of the organism in mg.
A_{gut}	0.07 cm^2	For Gammarus pulex, the gut was reported as of cylindrical shape with a diameter of 0.28-0.6 mm for animals of 10-15 mm length (Welton et al., 1983). Scaling down to a bodylength of 4.5 mm for *Hyalella azteca*, this amounts to a diameter of about 0.2 mm. The surface area of a cylinder of diameter 0.2 mm and length of approximately 4.5 mm amounts to 9.6*10^{-3} cm^2. Assuming the presence of villi in the gut, we multiply by a factor of 7.5 as is done for fish (Larisch & Goss, 2018a). Micro-villi have been observed in amphipods (Halcrow, 2001), we therefore apply a factor of 24 on the apical membrane permeability.<br>Note that this is a very rough estimation.
A_{skin}	0.14 cm^2	Total body surface area A_{skin} was roughly estimated, approximating the organism by a cylindrical shape of length 4.5 mm. Calculated with a density of 1 kg/L, the BW of 3 mg corresponds to a volume of 3 mm^3, and thus a cylindrical radius of 0.46 mm. This results in a total body surface area of 0.14 cm^2.

Table S2. Physiological data in fish: references and comments

	Fish<br>Used value	references and comments
Body composition		
Wet weight	2.2 g	Rainbow trout used in BCF studies taken from study of UV-329: https://echa.europa.eu/de/registration-dossier/-/registered-dossier/13220/5/4/2/?documentUUID=e0ac66f4- ba8f-461a-aaeb-0cd0f9f85aa2

Lipid content in organism $C_{L,org}$	0.037 kg_{lipid}/kg_{org}	Rainbow trout used in BCF studies taken from study of UV-329: https://echa.europa.eu/de/registration-dossier/-/registered-dossier/13220/5/4/2/?documentUUID=e0ac66f4- ba8f-461a-aaeb-0cd0f9f85aa2
Non Lipid Organic Matter content in organism $C_{NLOM,org}$	0.163 kg_{NLOM}/kg_{org}	calculated as the total content minus lipid and water content.
Water content in organism $C_{W,org}$	0.80 kg_{w}/kg_{org}	From (Naeem et al., 2016).
Respiration		
C_{OX}	11 mg O2/L_{w}	For 13°C, from (Benson & Krause, 1980).
temperature	13°C	As in study of UV-329: https://echa.europa.eu/de/registration-dossier/-/registered-dossier/13220/5/4/2/?documentUUID=e0ac66f4- ba8f-461a-aaeb-0cd0f9f85aa2
Ventilation rate constant	$2*10^{3}$ $L_{w}/kg_{org}/d$	Calculated using the empirical correlation (Arnot & Gobas, 2004): $$k_{vent} = \frac{1400 * BW^{-0.35}}{C_{OX}}$$ Where BW is the weight of the organism in kg, and C_{OX} the oxygen concentration in mg O_2/L_{w}.
Food		
feeding rate of organism G_d	0.01 $kg_{diet,wet}/d/kg_{org}$	1 - 2% of the mean body weight per day; taken from study of UV-329: https://echa.europa.eu/de/registration-dossier/-/registered-dossier/13220/5/4/2/?documentUUID=e0ac66f4- ba8f-461a-aaeb-0cd0f9f85aa2
dietary assimilation rate of lipids ε_L	92%	From (Arnot & Gobas, 2004) for fish
dietary assimilation rate of NLOM ε_N	60%	From (Arnot & Gobas, 2004) for fish
dietary assimilation rate of water ε_W	25%	From (Arnot & Gobas, 2004) for all fresh- water species
Blood		
Albumin-like protein concentration	41.2 g / L_{plasma}	Albumin protein concentration in fish from (Escher et al., 2011), assuming a protein density of 1.39 kg/L, this corresponds to 0.0296 $L_{albumin}/L_{plasma}$. The diffusion coefficient of albumin: $6.3*10^{-7}$ cm^2/s (Gaigalas et al., 1992).
Total plasma flow $k_{bf,tot}$	64 $L_{plasma}/kg/d$	Cardiac output Q_B was calculated as for fish after (Erickson & McKim, 1990): $Q_B = (0.23 * T - 0.78) * (\frac{M_{org}}{500})^{-0.1}$ with M_{org} in g and T in °C.

		To covert L_{blood} in L_{plasma}, Q_B was multiplied by 0.7 (Gingerich et al., 1987)
$r_{bf,gills}$	1	(Larisch et al., 2017)
$r_{bf,gut}$	0.178	(Larisch et al., 2017)
Organ surface areas		
A_{gills}	7 cm^2	(Morgan, 1971)
A_{gut}	3.8 cm^2	(Buddington & Diamond, 1987). Assuming the presence of villi in the gut, we multiplied by a factor of 7.5 (Larisch & Goss, 2018a).
A_{skin}	17 cm^2	A_{skin} /cm^2=10*(BW/g)$^{0.65}$ (Nichols et al., 1996)

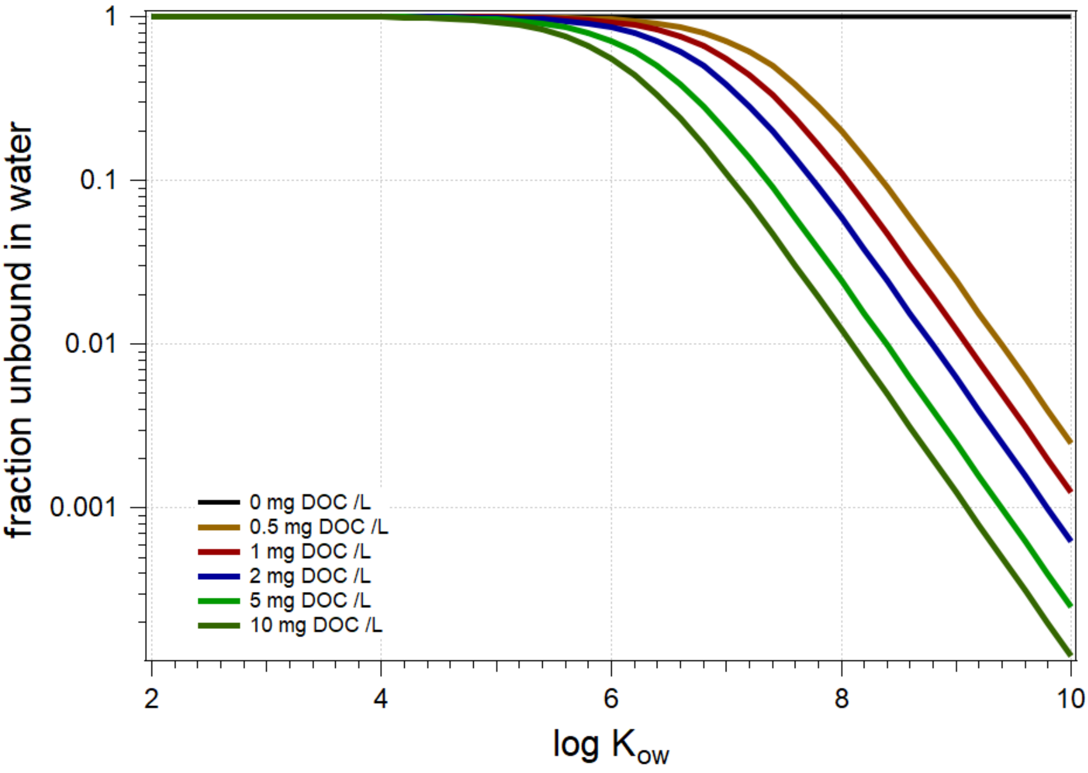

Figure S1. Fraction unbound (or bioavailable fraction) in water for the different indicated DOC concentrations in water. Trends predicted using Eq. 2.

S1 Diffusion

Diffusion is the driving force for the uptake (and elimination) of chemicals into the organism. Although borders between areas of diffusion and advection are not sharp in reality (Erickson & McKim, 1990), for simplicity we will strictly separate both processes. Diffusion will be deemed insignificant in areas dominated by water- or blood flow, and transport in the unstirred water layers or the membrane is assumed solely governed by diffusion. The diffusive flux is related to the concentration gradient by Fick's first law:

$$J = -D * \frac{dc}{dx} \tag{S1}$$

Where J is the diffusive flux (in chemical mass/area/time), D is the diffusion constant, c the chemical concentration and x the position. Diffusion constants in water were estimated from molecular weight (MW), according to (Avdeef, 2010):

$$D = 10^{-4.13-0.453*\log(MW)} \tag{S2}$$

Modeled compounds, molecular weight and calculated diffusion constants are listed in Table S3 and S4. We assumed equal diffusion coefficients in blood and water, but a reduction by a factor of 0.25 for the diffusion in the cytosol (Verkman, 2002).

Table S3: SMILES, Molecular weight MW and log K_{ow} of chemicals measured in *H. azteca*

Chemical	SMILES	MW (g/mol)	Log K_{ow}
UV-234	CC(C)(c1ccccc1)c(cc1C(C)(C)c2ccccc2)cc(c1O)n1 nc2ccccc2n1	447.6	7.29 - 9.84 [a, b]
UV-329	CC(C)(C)CC(C)(C)c(ccc1O)cc1n1nc2ccccc2n1	323.4	6.5 - 7.29 [a] 6.91 [c]
hexachlorobenzene	C1(=C(C(=C(C(=C1Cl)Cl)Cl)Cl)Cl)Cl	284.8	5.73 [d]
ortho-terphenyl	C1=CC=C(C=C1)C2=CC=CC=C2C3=CC=CC=C3	230.3	5.75 [d]
benzo(a)pyrene	C1=CC=C2C3=C4C(=CC2=C1)C=CC5=C4C(=CC=C 5)C=C3	252.3	6.13 [e]
PCB153	C1=C(C(=CC(=C1Cl)Cl)Cl)C2=CC(=C(C=C2Cl)Cl)Cl	360.9	6.34 [f]-7.75 [d,g]
PCB77	C1=CC(=C(C=C1C2=CC(=C(C=C2)Cl)Cl)Cl)Cl	292.0	6.72 [d]
chlorpyrifos	CCOP(=S)(OCC)OC1=NC(=C(C=C1Cl)Cl)Cl	350.57	4.96 [h]
methoxychlor	COC1=CC=C(C=C1)C(C2=CC=C(C=C2)OC)C(Cl)(Cl) Cl	345.6	5.08 [d]
pyrene	C1=CC2=C3C(=C1)C=CC4=CC=CC(=C43)C=C2	202.3	4.88 [d]
1,2,3-trichlorobenzene	C1=CC(=C(C(=C1)Cl)Cl)Cl	181.4	4.05 [h]
diazinon	CCOP(=S)(OCC)OC1=NC(=NC(=C1)C)C(C)C	304.3	3.81 [d]
simazine	CCNC1=NC(=NC(=N1)Cl)NCC	201.7	2.18 [d]
pentachlorobenzene	C1=C(C(=C(C(=C1Cl)Cl)Cl)Cl)Cl	250.3	5.18 [d]

Chemical	SMILES	MW (g/mol)	Log K_{ow}
azoxystrobin	CO/C=C(\C1=CC=CC=C1OC2=NC=NC(=C2)OC3=CC=CC=C3C#N)/C(=O)OC	403.4	2.5 [i]
prochloraz	CCCN(CCOC1=C(C=C(C=C1Cl)Cl)Cl)C(=O)N2C=CN=C2	376.7	4.1 [j]
terbutryn	CCNC1=NC(=NC(=N1)SC)NC(C)(C)C	241.4	3.74 [d]
trifloxystrobin	C/C(=N\OCC1=CC=CC=C1/C(=N/OC)/C(=O)OC)/C2=CC(=CC=C2)C(F)(F)F	408.4	4.5 [i]
methyl parathion	COP(=S)(OC)OC1=CC=C(C=C1)[N+](=O)[O-]	263.2	2.86 [d]
fluoranthene	C1=CC=C2C(=C1)C3=CC=CC4=C3C2=CC=C4	202.3	5.2 [d]
anthracene	C1=CC=C2C=C3C=CC=CC3=CC2=C1	178.2	4.45 [d]
dichlorodiphenyldichloroethylene (DDT)	C1=CC(=CC=C1C(=C(Cl)Cl)C2=CC=C(C=C2)Cl)Cl	318.0	6.51 [h]
fluorene	C1C2=CC=CC=C2C3=CC=CC=C31	166.2	4.18 [d]
phenanthrene	C1=CC=C2C(=C1)C=CC3=CC=CC=C32	178.2	4.46 [d]
dichlorodiphenyldichloroethane (DDD)	C1=CC(=CC=C1C(C2=CC=C(C=C2)Cl)C(Cl)Cl)Cl	320.0	6.02 [h]
dichlorodiphenyldichloroethylene (DDE)	C1=CC(=CC=C1C(=C(Cl)Cl)C2=CC=C(C=C2)Cl)Cl	318.0	6.51 [h]

[a] Predicted using various prediction methods
[b] Mean value 8.3±0.9 used in calculation, standard deviation used to determine model error bars
[c] Experimental (Do et al., 2022)
[d] Experimental (Hansch et al., 1995)
[e] Experimental (De Maagd et al., 1998)
[f] Experimental (Karickhoff et al., 1979)
[g] Mean value 7.05 used in calculation, min. and max. value used to determine model error bars
[h] Experimental J. Sangster: LogK_{ow} databank, version Jan. 1994
[i] Experimental (Tomlin, 2003)
[j] Experimental (Baker et al., 1992)

Table S4: Diffusion coefficient D_w, albumin/water partition coefficient $K_{albumin/w}$, aqueous solubility S_w, melting temperature T_m, subcooled solubility $S_{subcooled}$, calculated facilitation factors by bile micelles in the gut FAC_{mic} and albumin in blood $FAC_{ABL,albumin}$ for hydrophobic chemicals

Chemical	D_w [a] (cm^2/s)	Log $K_{albumin/w}$ [b]	S_w (mg/L)	T_m [c] (°C)	$S_{subcooled}$ [d] (mg/L)	FAC_{mic} [e]	$FAC_{ABL,albumin}$ [f]
UV-234	4.7E-06	7.31	1.65E-03 [g]	139	2.36E-02	125	3.2
UV-329	5.4E-06	6.05	1.68E-01 [g]	106	1.13E+00	11	1.3
hexachlorobenzene	5.73E-06	4.64	4.70E-03 [c]	229	5.29E-01	16	1.0
ortho-terphenyl	6.31E-06	4.84	1.24E+00 [c]	56	2.66E+00	5	1.0
benzo(a)pyrene	6.05E-06	5.15	1.62E-03 [c]	179	5.81E-02	53	1.0
PCB153	5.15E-06	6.05	9.49E-04 [c]	103	5.95E-03	247	1.2
PCB77	5.66E-06	5.35	1.80E-01 [c]	182	6.92E+00	4	1.1
chlorpyrifos	5.21E-06	3.40	1.12E+00 [c]	42	1.73E+00	9	1.0
methoxychlor	5.25E-06	4.63	9.99E-02 [c]	87	4.34E-01	20	1.0
pyrene	6.69E-06	4.40	1.35E-01[c]	151	2.55E+00	5	1.0
1,2,3-trichlorobenzene	7.03E-06	3.47	1.80E+01 [c]	51.3	3.45E+01	1	1.0
diazinon	5.56E-06	3.04	1.80E+01[c]	<25	<6.28 E+01	1	1.0
simazine	6.70E-06	1.45	6.00E+01 [c]	225	9.56E+02	1	1.0
pentachlorobenzene	6.07E-06	4.28	9.28E+00 [c]	86	3.53E+00	5	1.0
azoxystrobin	4.89E-06	2.25	8.31E-01 [c]	116	5.07E+01	1	1.0
prochloraz	5.05E-06	2.76	6.00E+00 [c]	48	6.04E+01	1	1.0
terbutryn	6.18E-06	2.79	3.40E+01 [c]	104	1.61E+02	1	1.0
trifloxystrobin	4.87E-06	4.36	2.51E+01 [c]	116	5.07E+01	1	1.0
methyl parathion	5.94E-06	2.22	6.00E+00 [c]	35.8	5.06E+01	1	1.0
fluoranthene	6.69E-06	4.42	3.77E+01 [c]	110.2	1.96E+00	6	1.0
anthracene	5.15E-06	6.05	2.65E-01c	103	5.95E-03	247	1.2
dichlorodiphenyldichloroethylene (DDT)	7.08E-06	3.94	9.49E-04[c]	216	3.64E+00	4	1.0
fluorene	5.45E-06	5.91	4.34E-02 [c]	89	2.96E-01	23	1.2
phenanthrene	7.31E-06	3.58	6.50E-02 [c]	114.8	1.39E+01	2	1.0

Chemical	D_w [a] (cm²/s)	Log $K_{albumin/w}$ [b]	S_W (mg/L)	T_m [c] (°C)	$S_{subcooled}$ [d] (mg/L)	FAC_{mic} [e]	$FAC_{ABL, albumin}$ [f]
dichlorodiphenyldichloroethane (DDD)	7.08E-06	3.94	1.69E+00 [c]	99	6.58E+00	3	1.0
dichlorodiphenyldichloroethylene (DDE)	5.43E-06	5.67	1.15E+00 [c]	109.5	6.55E-01	15	1.1

[a] predicted according to Eq. (S2)
[b] predicted using LSERD (Ulrich et al., 2017)
[c] experimental values taken from Pubchem https://pubchem.ncbi.nlm.nih.gov/
[d] predicted using Eq. (S10)
[e] predicted using Eq. (S9)
[f] predicted for ABL blood thickness of 286 nm and albumin mass fraction of 41.2 g/L_{plasma},
[g] predicted using Episuite.

S2 Facilitation factor in ABL_{blood}

For the calculation of the facilitation factor in blood, albumin-like proteins with similar binding properties were assumed to be present in *H. azteca* at the same concentration as in fish. Albumin/water partition coefficients were either calculated with LSERD (Ulrich et al., 2017) using experimental descriptors, or the following correlation with the log K_{ow} (Endo & Goss, 2011):

$$K_{ALB/water} = 0.71 * \log K_{ow} + 0.42 \quad \text{(S3)}$$

The desorption rate constant from albumin was calculated from an empirical correlation with the chemical's molecular weight according to Eq. (S4) (Krause et al., 2018):

$$k_{des,ALB} = 20267 * MW_{chemical}^{-2} \quad \text{(S4)}$$

Where $MW_{chemical}$ is the chemical's molecular weight in g/mol, and k_{des} is the desorption rate constant in 1/s.

The relation between solute bound to albumin $m_{bound,ALB}$ and solute freely dissolved in water m_{free} in equilibrium can be expressed as:

$$\frac{m_{bound,ALB}}{m_{free}} = \frac{k_{sorb,ALB}}{k_{des,ALB}} = K_{ALB/water} * c_{ALB} \quad \text{(S5)}$$

Where $K_{ALB/water}$ is the albumin/water partition coefficient, c_{ALB} is the concentration of albumin in water, and $k_{sorb,ALB}$ is the sorption rate constant to albumin in 1/s.

$$k_{sorb,ALB} = K_{ALB/water} * c_{ALB} * k_{des,ALB} \quad \text{(S6)}$$

To calculate the influence of facilitated transport by the carrier albumin in blood, compound both freely dissolved in water and bound to albumin must be considered separately. Only the unbound fraction can move across cell membranes, but both fractions may traverse the unstirred water layer, see scheme in Figure S2 on the right.

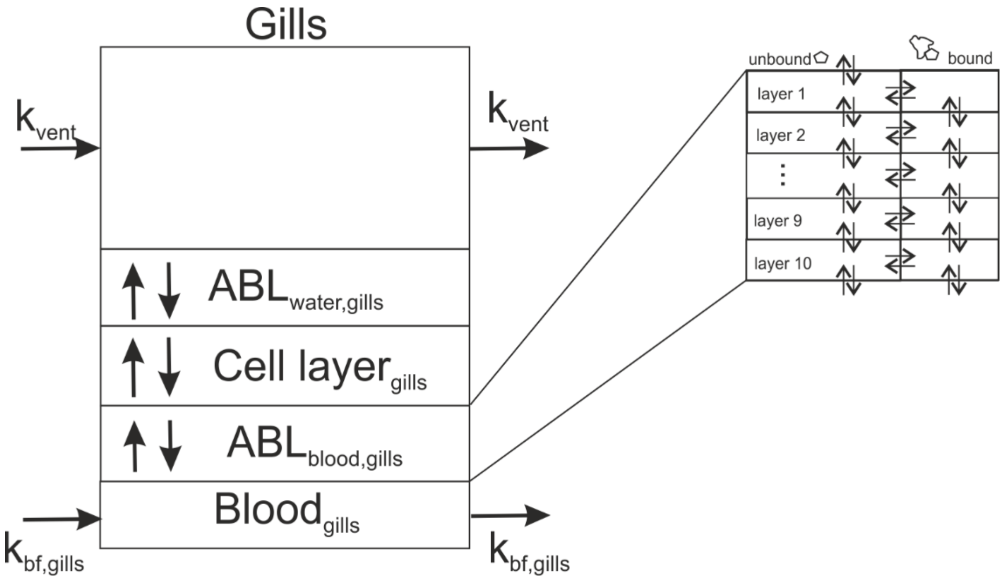

Figure S2 Diffusion steps in gills (left) and facilitated transport of the chemical across the ABL in blood (right). Vertical arrows on the right depict diffusion of the chemical (unbound and bound to the carrier), horizontal arrows de-/sorption processes from/to the carrier. Only the unbound species can traverse the cell membrane and enter layer 1 from the membrane side, but both can traverse into the gill blood compartment ($Blood_{gills}$), hence the asymmetrical depiction of arrows.

Transport rate constants for the diffusion through the ABL in blood differ from the free chemical, because the transport of the bound chemical is limited by the diffusion of the carrier:

$$k_{ABL,bound} = \frac{D_{carrier} * A}{d_{ABL}} / M_{org} \quad \text{(S7)}$$

Where $D_{carrier}$ is the diffusion constant of the carrier, in this case albumin.

We separated the ABL into 10 layers. Sorption kinetics are slow in relation to the residence time in the respective layers, we are thus limited by de-/sorption kinetics. Sorption processes will take place during the whole diffusion process, but a molecule only carried the last little stretch of the way by a carrier will not contribute the same way to the facilitation factor as a molecule picked up from the start. For an ABL of 286 nm, the arising difference between 1 and 10 layers in the calculations amounted to a factor of about 10.

For the unbound and bound chemical, mass balance equations can be set up in each layer, as each form can move from or to the neighboring layer, or sorb or desorb from albumin in the respective layer. Solving the system of equations in Table S5 via the Gauss algorithm, we get the rate constant considering facilitated transport. Comparing this rate constant to the one without considering facilitated transport, we can calculate FAC_{ABL}.

The resulting facilitation factors FAC_{ABL} are then used to calculate the rate constant of diffusion through the ABL in blood:

$$k_{ABL,blood} = FAC_{ABL} * k_{ABL,blood,unbound} = FAC_{ABL} * \frac{D_w * A}{d_{ABL}} / M_{org} \quad (S8)$$

Where D_w is the diffusion constant of the chemical in blood (assumed equal to water) and d_{ABL} the total thickness of the ABL.

Table S5: System of equations considering de-/sorption kinetics to calculate FAC in blood.

Equation	Cabl_blood, unbound, layer1	Cabl_blood,bound, layer1	Cabl_blood, unbound, layer2	Cabl_blood, bound,layer2	layers 3…10	Cblood,unbound	Cblood,bound	
I	$- k_x - k_{ABL,blood,unbound} - k_{sorb} * V_{blood\ layer} / M_{org}$	$+ k_{des} * V_{layer} / M_{org}$	$+ k_{ABL,blood,unbound}$	0	…	0	0	$- k_x * c_{w,unbound}$
II	$+ k_{sorb} * V_{layer} / M_{org}$	$- k_{ABL,blood,bound} - k_{des} * V_{layer} / M_{org}$	0	$+ k_{ABL,blood,bound}$	…	0	0	0
III	$+ k_{ABL,blood,unbound}$	0	$- 2 * k_{ABL,blood,unbound} - k_{sorb} * V_{layer} / M_{org}$	$+ k_{des} * V_{layer} / M_{org}$	…	0	0	0
IV	0	$+ k_{ABL,blood,bound}$	$+ k_{sorb} * V_{layer} / M_{org}$	$- 2 * k_{ABL,bound} - k_{des} * V_{layer} / M_{org}$	…	0	0	0
V-XX	…	…	…	…	…	…	…	…
XXI	0	0	0	0	…	$- k_{ABL,blood,unbound} - k_{sorb} * V_{ABL\_blood} / M_{org}$	$+ k_{des} * V_{blood} / M_{org}$	0
XXII	0	0	0	0	…	$+ k_{sorb} * V_{blood} / M_{org}$	$- k_{ABL,bound} - k_{des} * V_{blood} / M_{org}$	0

$V_{layer} = A * d_{ABL,blood,layer} * 0.7 = A * d_{ABL,blood} / 10 * 0.7$ the volume of a single ABL-layer, $k_{ABL,blood,unbound}$/ $k_{ABL,blood,bound}$ rate constant for diffusion in the ABL in blood for freely dissolved chemical/chemical bound to albumin-like proteins for an ABL layer of thickness $d_{ABL}/10$, k_x rate constant for diffusion from water to the first layer of the ABL (combines all preceding diffusion steps, for simplicity), $c_{w,unbound}$ is the chemical concentration in water of the unbound chemical. V_{blood} is the volume of the organ blood pool, which is assumed well mixed and is big enough for the bound and unbound chemical to be in equilibrium with each other. The factor 0.7 is used to transform blood volume to plasma volume.

S3 Bile acids as carriers in the gut

The facilitated transport via bile acids in the gut was estimated from subcooled solubility according to (Larisch, 2019; Larisch & Goss, 2018b; Westergaard & Dietschy, 1976):

$$FAC_{mic} = 0.3972 * S_{subcooled}^{-0.584} \quad \text{(S9)}$$

Where $S_{subcooled}$ is the subcooled solubility in mM. It is calculated approximatively according to (Liu et al., 2013) from aqueous solubility S_w and melting temperature T_m (in K):

$$S_{subcooled} = \frac{S_w}{\exp[\frac{T_m * \Delta S_m}{R * T_m} * (1 - \frac{T_m}{T})]} / MW \quad \text{(S10)}$$

With T being the temperature in Kelvin, R the gas constant, and ΔS_m the entropy of fusion with approximately 56.5 J/mol/K. The aqueous solubility S_w (in mg/L) was predicted using EPIsuite (US EPA. 2012 Estimation Programs Interface Suite™ for Microsoft® Windows, v 4.1. United States Environmental Protection Agency, Washington, DC, USA.) if no experimental value was available.

Note the uncertainty of these predictions, because the prediction model of the FAC_{mic} by micellar transport is an empirical model based solely on fatty acids as input data. Although the FAC depends on log K_{ow}, there is wide scatter, see Figure S3. Thus, the estimated FAC depending on log K_{ow} can only be considered as a rough estimate, and specific FACs should be calculated for specific chemicals for better results. For resulting FAC below 1, FAC was set to 1.

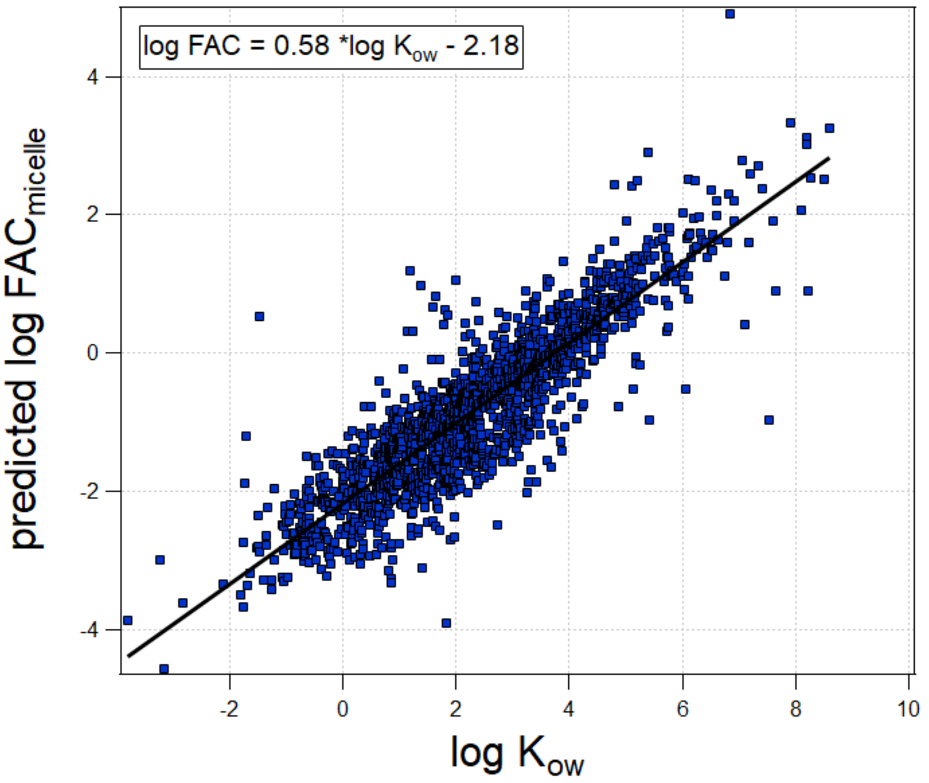

Figure S3 Correlation between calculated log FAC and experimental log K_{ow}. Micelle facilitation factors were calculated at 297 K using Eq. (**S9**) and (**S10**) for 1601 chemicals for which experimental log K_{ow} (Mansouri et al., 2016), experimental water solubilities and experimental melting temperatures were available (QSAR Toolbox version 4.4.1, which is freely available on the OECD website (http://www.qsartoolbox.org/)). Trendline: log FAC =0.58*log K_{ow} - 2.18.

Table S6. Calculated facilitation factors by bile micelles in the gut FAC_{mic} and albumin in blood $FAC_{ABL,albumin}$ depending on the octanol/water partition coefficient log K_{ow}

Log K_{ow}	FAC_{mic} [a]	$FAC_{albumin}$ [b]	Log K_{ow}	FAC_{mic} [a]	$FAC_{ABL,albumin}$ [b]
1.0	1	1.0	5.6	12	1.0
1.2	1	1.0	5.8	15	1.0
1.4	1	1.0	6.0	20	1.0
1.6	1	1.0	6.2	26	1.0
1.8	1	1.0	6.4	34	1.0
2.0	1	1.0	6.6	44	1.0
2.2	1	1.0	6.8	58	1.0
2.4	1	1.0	7.0	76	1.1
2.6	1	1.0	7.2	99	1.1
2.8	1	1.0	7.4	129	1.1
3.0	1	1.0	7.6	169	1.1
3.2	1	1.0	7.8	221	1.2
3.4	1	1.0	8.0	288	1.3
3.6	1	1.0	8.2	377	1.4
3.8	1	1.0	8.4	492	1.5
4.0	1	1.0	8.6	643	1.7
4.2	2	1.0	8.8	839	2.0
4.4	2	1.0	9.0	1096	2.3
4.6	3	1.0	9.2	1432	2.7
4.8	4	1.0	9.4	1871	3.2

Log K_{ow}	FAC_{mic} [a]	$FAC_{albumin}$ [b]		Log K_{ow}	FAC_{mic} [a]	$FAC_{ABL,albumin}$ [b]
5.0	5	1.0		9.6	2443	3.8
5.2	7	1.0		9.8	3192	4.6
5.4	9	1.0		10	4169	5.6

[a] predicted using Eq. (S9), [b] predicted for ABL blood thickness of 286 nm and albumin mass fraction of 41.2 g/L_{plasma}

S4 Cell permeation

Although permeability through cell membranes correlates more strongly with hexadecane/water than with octanol/water partition coefficients (Walter & Gutknecht, 1986), for neutral compounds we will for simplicity use the empirical correlation between membrane permeability P_{mem} and the octanol/water partition coefficient to calculate P_{mem} (Walter & Gutknecht, 1986):

$$\log P_{mem} = 1.15 * log\ K_{ow} - 2.14 \quad \text{(S11)}$$

With P_{mem} in cm/s, and P_{mem}=1/ R_{mem}. For the P_{mem} of the apical gut membrane, we multiply a factor of 24 to account for the presence of microvilli in the gut (Halcrow, 2001). Lateral transport and cytosolic transport are calculated as described in detail in (Bittermann & Goss, 2017).

Table S7 Chemical name, experimental aqueous exposure concentration c_{exp}, experimental rate constants k1 and k2 and respective errors, experimental whole body metabolic rate constant $k_{m,exp}$, experimental kinetic log BCF and respective error from several literature sources as indicated.

Chemical [a]	c_{exp} (µg/L_w)	$k1_{exp}$ (L_w/kg_{org}/d)	Error_$k1_{exp}$ (L_w/kg_{org}/d)	$k2_{exp}$ (1/d)	Error_$k2_{exp}$ (1/d)	$k_{m,exp}$ (1/d)	Log BCF_{exp} [b]	Error_ log BCF_{exp}
Hexachlorobenzene [c]	0.60	2.75E+03	3.56E+02	4.17E-01	6.40E-02		4.41	0.95
ortho-terphenyl [c]	1	1.22E+03	8.00E+01	4.65E-01	8.20E-02		4.01	0.81
benzo(a)pyrene [c]	4	6.66E+03	4.00E+02	2.04E+00	9.80E-02		3.81	0.42
1,2,3-trichlorobenzene [c]	15	2.64E+02	1.20E+02	2.31E+01	7.63E+00		1.66	0.99
PCB153 [c]	30	7.88E+03	3.31E+03	7.90E-02	3.00E-03		5.41	0.65
PCB77 [c]	10	6.62E+03	3.47E+02	1.64E-01	6.00E-03		5.01	0.63
diazinon [c]	40	8.64E+02	2.16E+02	3.65E+01	2.44E+01		1.91	1.47
chlorpyrifos [c]	20	4.34E+02	5.90E+01	4.73E-01	2.47E-01		3.29	1.81
methoxychlor [c]	30	4.95E+03	3.61E+02	7.98E-01	1.12E-01		3.79	0.60
pyrene [c]	40	4.19E+03	2.01E+02	7.14E-01	5.20E-02		3.77	0.33

Chemical [a]	c_{exp} (µg/L_w)	$k1_{exp}$ (L_w/kg_{org}/d)	Error_$k1_{exp}$ (L_w/kg_{org}/d)	$k2_{exp}$ (1/d)	Error_$k2_{exp}$ (1/d)	$k_{m,exp}$ (1/d)	Log BCF_{exp} [b]	Error_ log BCF_{exp}
simazine [c]	6	4.80E+00	1.85E+01	1.03E+00	1.92E-01		1.18	0.38
Pentachlorobenzene [d]	4.8	1.08E+03	3.14E+02	5.52E-01	1.68E-01		3.69	
azoxystrobin [e]	113	4.75E+00	3.77E+01	1.19E+00	3.30E-01	1.8	0.95	
prochloraz [e]	55	4.22E+02	1.36E+01	4.35E+00	5.70E-01	1.6	2.49	
terbutryn [e]	55	2.47E+02	9.90E+00	6.64E+00	2.20E-01	6.1	1.89	
trifloxystrobin [e]	0.70	6.20E+02	4.92E+01	1.58E+00	2.20E-01	1.8	2.98	
methyl parathion [f]	2.30	2.76E+02	2.64E+01	2.26E+01	1.68E+00	12.9	1.48	
fluoranthene [f]	45,1	4.43E+03	2.23E+02	2.11E+00	1.92E-01	1.15	3.72	
PCB153 [f]	3	6.04E+03	2.16E+02	9.60E-02	2.40E-02		5.20	
chlorpyrifos [g]	4	3.52E+04	2.90E+03	6.22E+00			4.15	
anthracene [h]	7.7-8.7	6.12E+03	1.82E+03	3.84E+00	7.20E-01	0.576	3.60	
dichlorodiphenyldichloroethylene (DDT) [i]	0.61	7.21E+03	1.23E+03	1.51E-01	3.60E-02		5.08	
fluoroanthene [j]	15.6	1.98E+03	3.96E+02	1.66E+00	1.49E+00		3.48	
fluorene [k]	295	5.98E+03	1.42E+03	2.25E+01	5.59E+00		2.82	
phenanthrene [k]	87.00	3.41E+03	2.64E+02	7.75E+00	6.24E-01		3.04	
dichlorodiphenyldichloroethylene (DDT) [l]	0.02	6.46E+03	1.01E+03	2.38E-01	2.40E-02		4.83	
dichlorodiphenyldichloroethane (DDD) [l]	0.18	8.03E+03	2.59E+03	4.80E-01	5.76E-02		4.62	
dichlorodiphenyldichloroethylene (DDE) [l]	1.12	1.30E+04	2.68E+03	2.95E-01	1.68E-02		5.04	
pyrene [k]	9.40	1.57E+04	1.22E+03	3.05E+00	2.88E-01		4.11	
UV-329 [m]	0.21	8.29E+03		9.92E-01			4.30	
UV-234 [m]	1	1.98E+02		2.73E-01			3.18	

[a] Some chemicals are stated several times, if they were measured in different publications. Duplicates within the same publication are not listed here. Lowest concentrations and longest experimental duration were selected preferably, as stated in the methods section.
[b] If available taken directly from literature, otherwise calculated dividing k1/k2 and lipid normalized to 5%, assuming a lipid concentration in the organism of 2%. Kinetic BCF were used, since not for all experiments steady state values were stated, and not all experiments reached steady state.
[c] (Schlechtriem et al., 2019), 23±3°C, 28 days old *H. azteca*
[d] (Landrum et al., 2004), 20-22°C, 7-10 days old *H. azteca*
[e] (Kosfeld et al., 2020), 25±2°C, >2 months old *H. azteca*
[f] (Nuutinen et al., 2003), 21±1°C, 2-3 weeks old *H. azteca*

[g] (Johanif et al., 2021), 23°C, 14-21 days old *H. azteca*
[h] (Landrum & Scavia, 1983), 21-25°C
[i] (Landrum et al., 2005), 23±1°C, 10-14 days old *H. azteca*
[j] (Schuler et al., 2004), 23°C
[k] (Lee et al., 2002), 23°C, 2-3 weeks old *H. azteca*
[l] (Lotufo et al., 2000), 18.2-21°C, 1-2 weeks old *H. azteca*
[m] (Schlechtriem et al., 2022), 25±2°C, >2 months old *H. azteca*

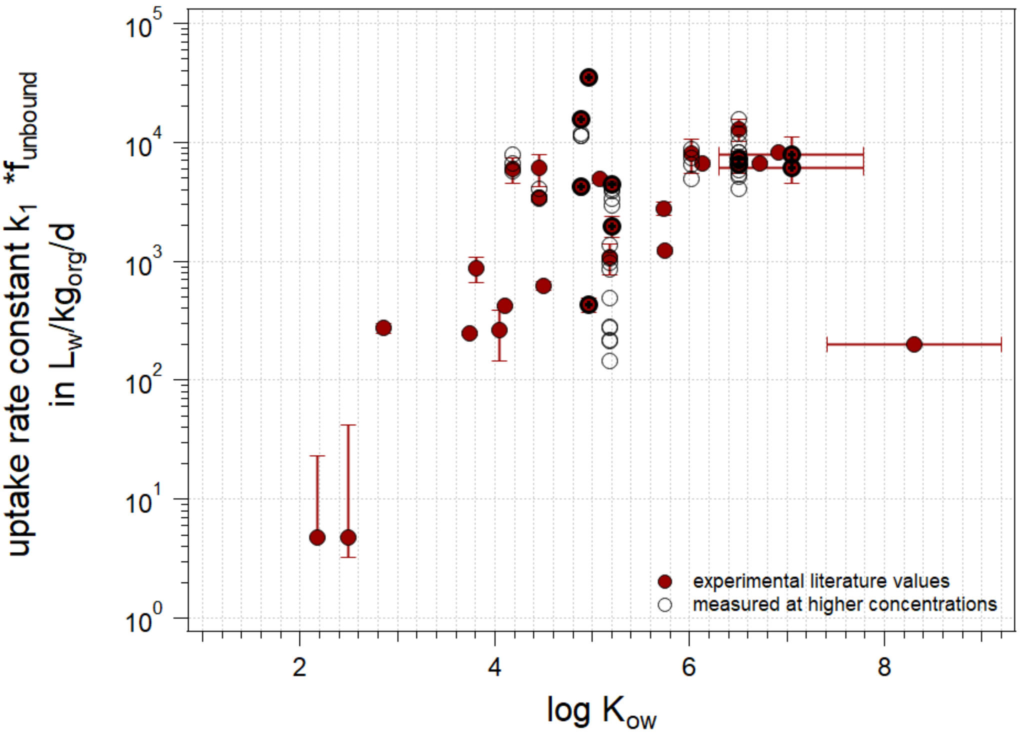

Figure S4 Influence of different chemical concentrations in the exposure medium on k1.

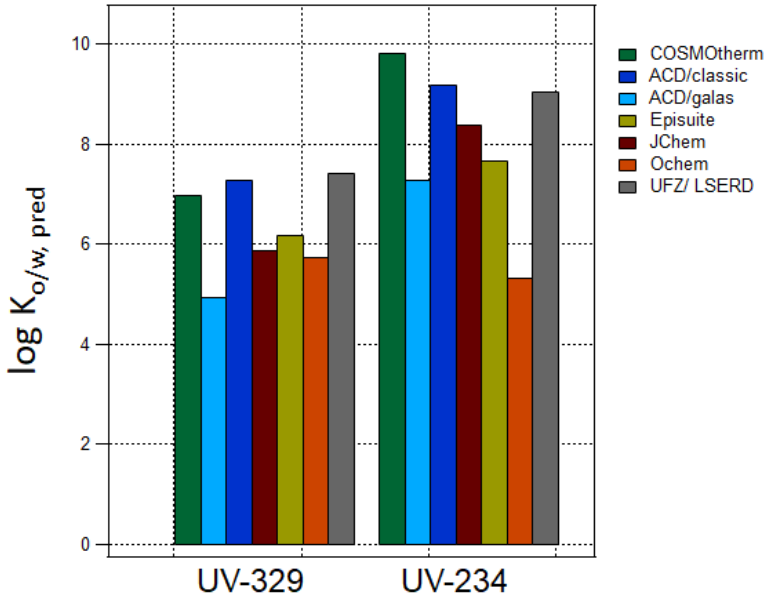

Figure S5 Octanol/water partition coefficients predicted for UV-329 and UV-234 using different prediction tools. Predictions were done with COSMOtherm (COSMOtherm, Release 18. COSMOlogic, GmbH & Co. KG, Leverkusen, Germany. http://www.cosmologic.de.) (Eckert & Klamt, 2002), ACD/classics and ACD/Galas (ACD Percepta (2015 Release)), KOWWIN in EPI-SUITE (EPI-Suite. US EPA Estimation Programs Interface Suite™ for Microsoft® Windows, v 411. (https://www.epa.gov/tsca-screening-tools/epi-suitetm- estimation-program-interface) (United States Environmental Protection Agency, 2012)),JChem for Excel (ChemAxon. JChem for Excel v. 20.6.0.618. https://chemaxon.com/ (2020).), Ochem (Sushko et al., 2011), and UFZ LSERD(Ulrich et al., 2017). For UV-329, a recently determined experimental value of 6.91 was finally used in the modeling (Do et al., 2022).

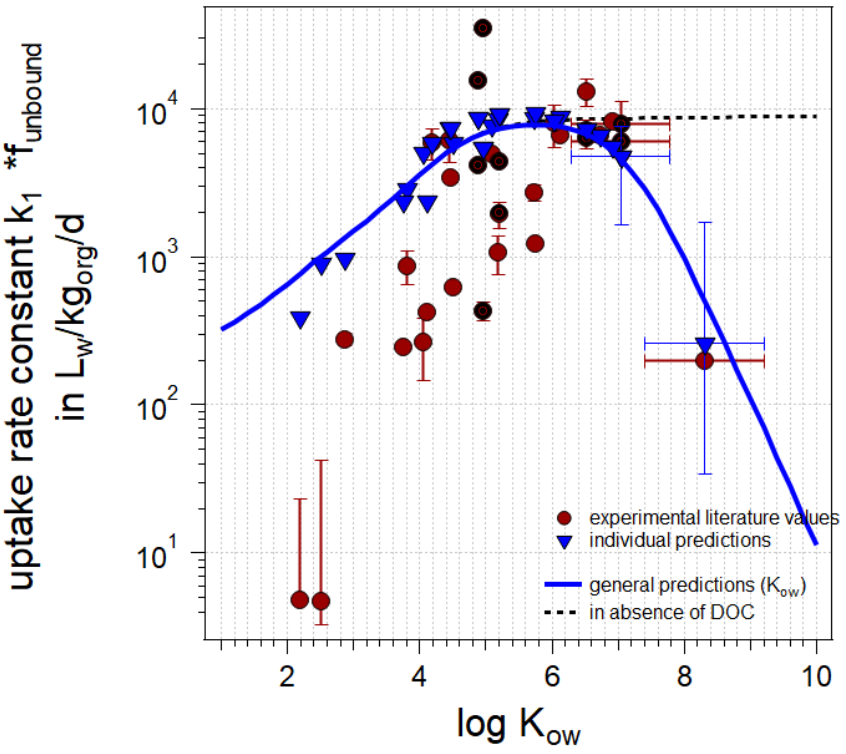

Figure S6 Predicted k1 according to Eq. (3) for blood flow as in fish. k1 values of same chemicals taken from different literature are marked with a cross.

Table S8 Chemical name, experimental uptake rate constant k1 considering 1 mg DOC /L_w and elimination rate constant $k_{2,gills}$+$k_{2,gut}$ (considering ventilation and feces) and k2 (considering ventilation, feces and metabolism), log BCF and log BCF_m not considering/considering metabolism respectively, predicted metabolic rate constant, predicted metabolic half-life. Blood flow is assumed as in fish.

Chemical	k1 (L_w/kg_{org}/d)	$k_{2,gills}$+$k_{2,gut}$ (1/d)	k2 (1/d)	Log BCF	Log BCFm	$k_{m,pred}$ (1/d)	half-life (h)[a]
UV-234	2.61E+02	7.04E-03	1.06E-01	4.97	3.79	9.89E-02	168.16
UV-329	5.63E+03	6.16E-02	5.76E-01	5.36	4.39	5.14E-01	32.33
hexachlorobenzene	8.72E+03	6.40E-01	6.96E-01	4.53	4.50	5.62E-02	296.03
ortho-terphenyl	9.41E+03	6.55E-01	8.42E-01	4.56	4.45	1.87E-01	88.78
benzo(a)pyrene	8.85E+03	3.03E-01	5.98E-01	4.86	4.57	2.95E-01	56.38
PCB153	4.76E+03	7.37E-02	7.53E-02	5.21	5.20	1.58E-03	10522.54
PCB77	6.68E+03	7.75E-02	8.95E-02	5.33	5.27	1.20E-02	1387.17
chlorpyrifos	5.49E+03	2.17E+00	2.25E+00	3.80	3.79	8.27E-02	201.23
methoxychlor	7.81E+03	2.34E+00	2.43E+00	3.92	3.90	8.99E-02	185.02
pyrene	8.81E+03	4.13E+00	4.41E+00	3.73	3.70	2.81E-01	59.24
1,2,3-trichlorobenzene	5.10E+03	1.59E+01	1.61E+01	2.90	2.90	1.77E-01	93.73
diazinon	2.87E+03	1.55E+01	1.58E+01	2.66	2.66	2.57E-01	64.7
simazine	3.95E+02	7.82E+01	7.89E+01	1.10	1.10	6.84E-01	24.3

Chemical	k1 ($L_w/kg_{org}/d$)	$k_{2,gills}+k_{2,gut}$ (1/d)	k2 (1/d)	Log BCF	Log BCFm	$k_{m,pred}$ (1/d)	half-life (h)[a]
pentachlorobenzene	8.54E+03	2.04E+00	2.12E+00	4.02	4.00	7.72E-02	215.37
azoxystrobin	9.02E+02	9.23E+01	9.41E+01	1.39	1.38	3.66E+00	4.54
prochloraz	2.36E+03	6.59E+00	8.19E+00	2.95	2.86	2.52E-01	66.04
terbutryn	2.37E+03	1.50E+01	2.11E+01	2.60	2.45	2.28E+00	7.28
trifloxystrobin	5.91E+03	6.59E+00	8.39E+00	3.35	3.25	1.51E+00	11.05
methyl parathion	9.67E+02	4.51E+01	5.81E+01	1.73	1.62	1.03E+00	16.07
fluoranthene	9.32E+03	2.13E+00	3.28E+00	4.04	3.85	3.02E-01	55.14
anthracene	7.41E+03	9.25E+00	9.83E+00	3.30	3.28	1.58E+00	10.54
dichlorodiphenyldichloro ethylene (DDT)	7.38E+03	1.41E-01	1.63E-01	5.12	5.05	2.16E-02	769.62
fluorene	5.93E+03	1.37E+01	1.39E+01	3.03	3.03	1.76E-01	94.64
phenanthrene	7.44E+03	9.08E+00	9.58E+00	3.31	3.29	4.99E-01	33.32
dichlorodiphenyldichloro ethane (DDD)	8.44E+03	3.51E-01	3.61E-01	4.78	4.77	1.05E-02	1578.56
dichlorodiphenyldichloro ethylene (DDE)	7.38E+03	1.43E-01	1.65E-01	5.11	5.05	2.16E-02	769.62

[a] Biotransformation half-life was predicted using (Brown et al., 2012; EAS-E Suite, 2022).

Table S9 Chemical name, experimental uptake rate constant k1 considering 1 mg DOC /Lw and elimination rate constant $k_{2,gills}+k_{2,gut}$ (considering ventilation and feces) and k2 (considering ventilation, feces and metabolism), log BCF and log BCF_m not considering/considering metabolism respectively, predicted metabolic rate constant, predicted metabolic half-life. Blood flow is assumed reduced by a factor of 20 as compared to fish.

Chemical	k1 ($L_w/kg_{org}/d$)	$k_{2,gut}$ (1/d)	k2 (1/d)	Log BCF	Log BCF_m	$k_{m,pred}$ (1/d)	half-life (h)[a]
UV-234	2.61E+02	6.74E-03	1.06E-01	4.99	3.79	9.89E-02	168.16
UV-329	5.52E+03	5.94E-02	5.74E-01	5.37	4.38	5.14E-01	32.33
hexachlorobenzene	5.70E+03	4.26E-01	4.82E-01	4.53	4.47	5.62E-02	296.03
ortho-terphenyl	6.91E+03	4.87E-01	6.74E-01	4.55	4.41	1.87E-01	88.78
benzo(a)pyrene	7.52E+03	2.57E-01	5.52E-01	4.86	4.53	2.95E-01	56.38
PCB153	4.66E+03	6.61E-02	6.77E-02	5.25	5.24	1.58E-03	10522.54
PCB77	6.03E+03	6.96E-02	8.16E-02	5.34	5.27	1.20E-02	1387.17
chlorpyrifos	8.42E+02	3.53E-01	4.36E-01	3.78	3.68	8.27E-02	201.23
methoxychlor	5.31E+03	1.61E+00	1.70E+00	3.92	3.89	8.99E-02	185.02

Chemical	k1 ($L_w/kg_{org}/d$)	$k_{2,gut}$ (1/d)	k2 (1/d)	Log BCF	Log BCF_m	$k_{m,pred}$ (1/d)	half-life (h)[a]
pyrene	4.65E+03	2.20E+00	2.48E+00	3.72	3.67	2.81E-01	59.24
1,2,3-trichlorobenzene	9.54E+02	3.01E+00	3.19E+00	2.90	2.87	1.77E-01	93.73
diazinon	3.85E+02	2.12E+00	2.38E+00	2.66	2.61	2.57E-01	64.7
simazine	2.29E+01	4.64E+00	5.33E+00	1.09	1.03	6.84E-01	24.3
pentachlorobenzene	3.97E+03	9.69E-01	1.05E+00	4.01	3.98	7.72E-02	215.37
azoxystrobin	7.62E+01	7.87E+00	9.67E+00	1.38	1.29	3.66E+00	4.54
prochloraz	2.17E+02	6.38E-01	2.24E+00	2.93	2.39	2.52E-01	66.04
terbutryn	2.31E+02	1.51E+00	7.61E+00	2.58	1.88	2.28E+00	7.28
trifloxystrobin	3.57E+03	3.99E+00	5.79E+00	3.35	3.19	1.51E+00	11.05
methyl parathion	7.27E+01	3.45E+00	1.64E+01	1.72	1.04	1.03E+00	16.07
fluoranthene	4.88E+03	1.13E+00	2.28E+00	4.03	3.73	3.02E-01	55.14
anthracene	2.35E+03	2.96E+00	3.53E+00	3.30	3.22	1.58E+00	10.54
dichlorodiphenyldichloro ethylene (DDT)	7.18E+03	1.36E-01	1.57E-01	5.12	5.06	2.16E-02	769.62
fluorene	1.20E+03	2.82E+00	3.00E+00	3.03	3.00	1.76E-01	94.64
phenanthrene	2.35E+03	2.90E+00	3.40E+00	3.31	3.24	4.99E-01	33.32
dichlorodiphenyldichloro ethane (DDD)	8.04E+03	3.34E-01	3.45E-01	4.78	4.77	1.05E-02	1578.56
dichlorodiphenyldichloro ethylene (DDE)	7.18E+03	1.38E-01	1.59E-01	5.12	5.05	2.16E-02	769.62

[a] Biotransformation half-life was predicted using (Brown et al., 2012; EAS-E Suite, 2022).

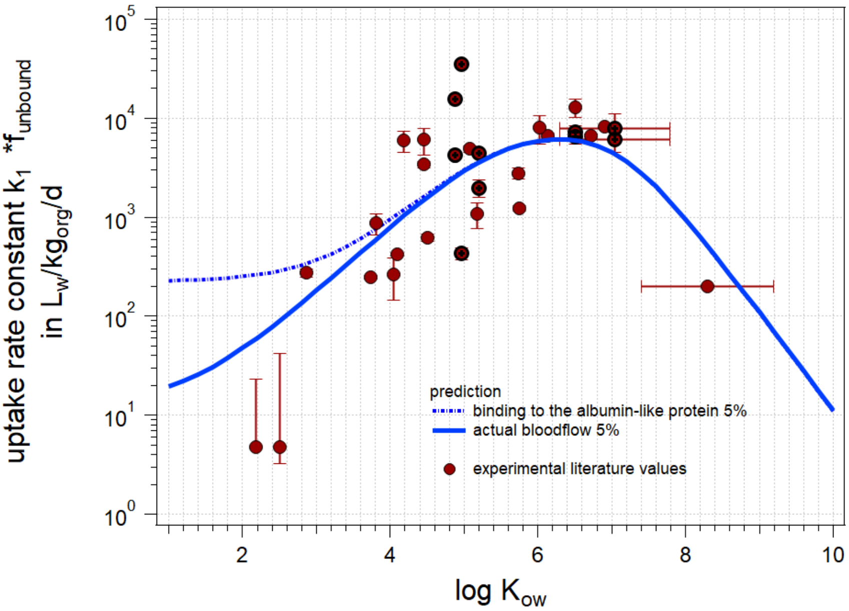

Figure S7 Differences in modeled k1 if transport by blood flow is reduced due to a reduced binding to albumin and thus a reduction in sorption capacity of the blood, or by an actual reduction in blood flow.

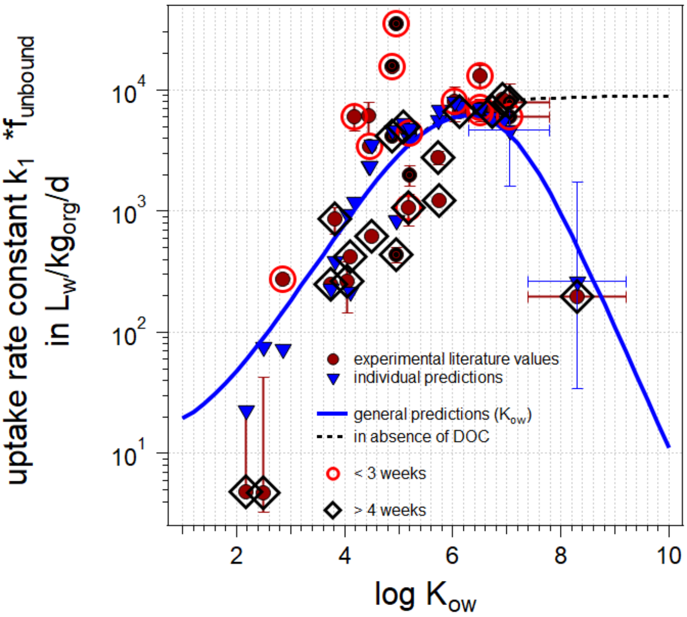

Figure S8 Differences in k1 due to organism age at the start of the experiment. Young, immature amphipods tend to have higher k1 than mature ones. If a datapoint is not marked, no age was available in the respective publication.

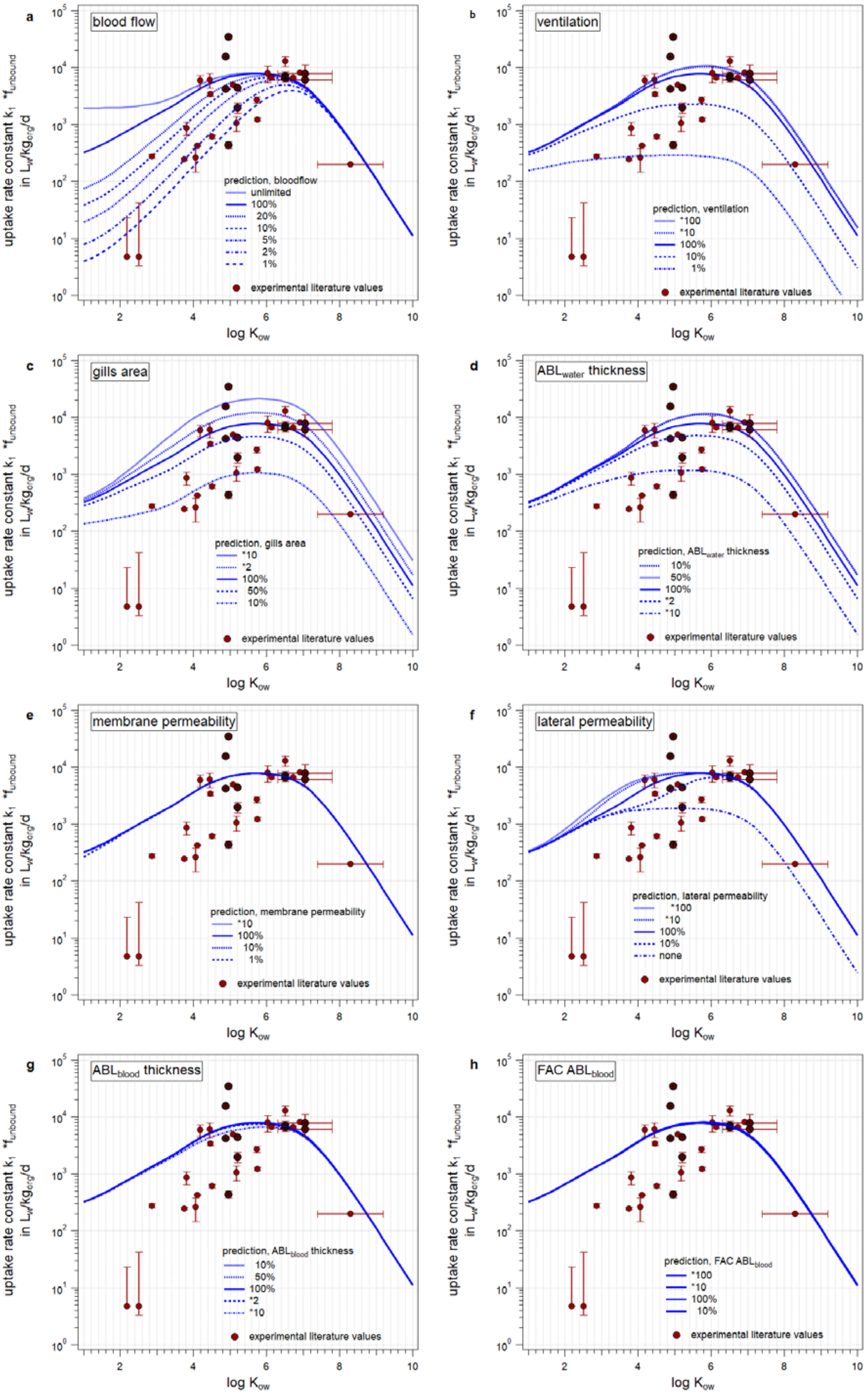

Figure S9 Sensitivity analysis in *H. azteca*, blood flow calculated as in fish.

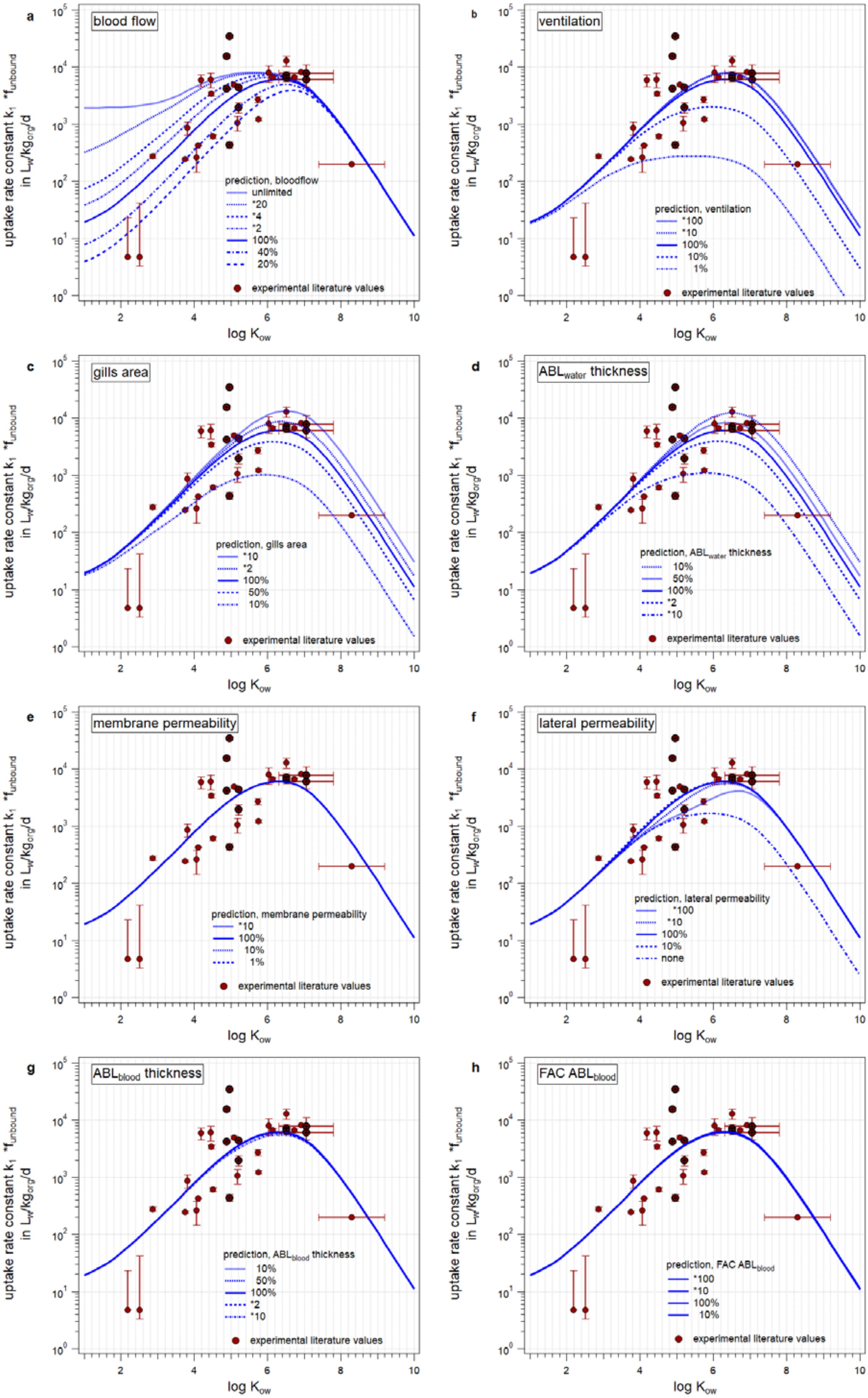

Figure S10. Sensitivity analysis in *H. azteca*, calculated with adapted blood flow.

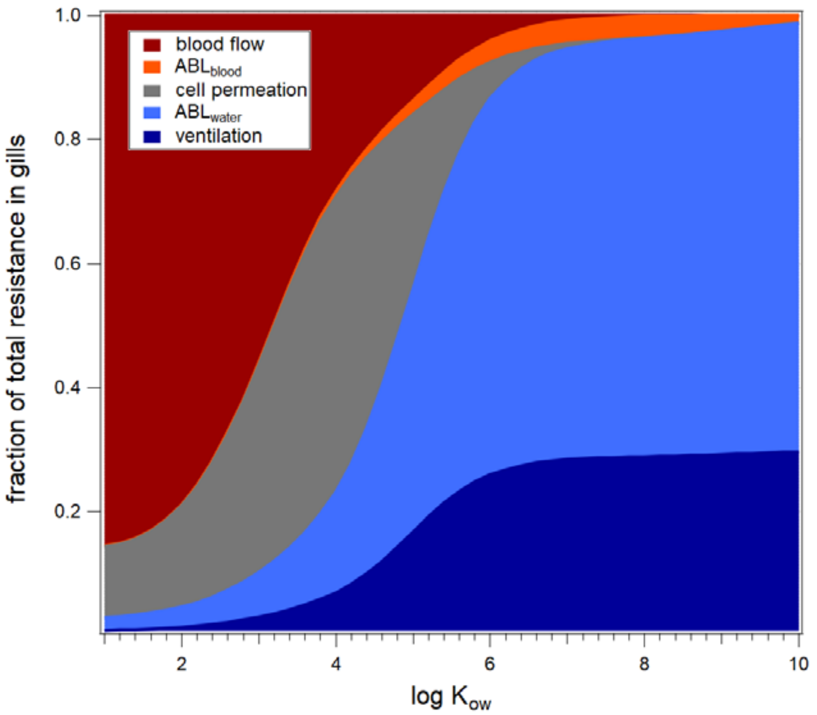

Figure S11. Main resistances for uptake via gills in *H. azteca* if blood flow is calculated as in fish.

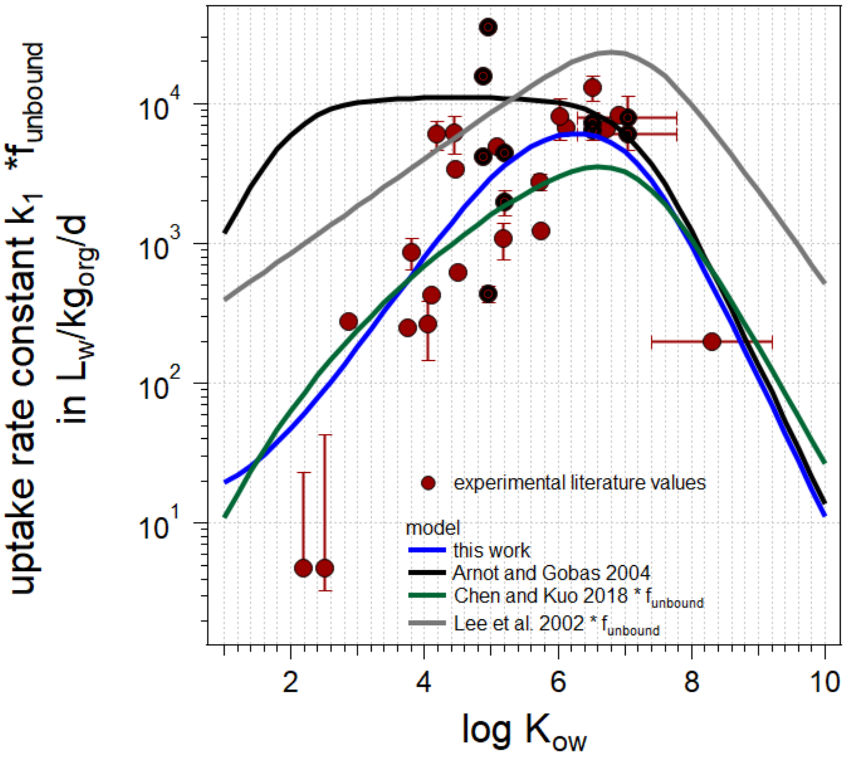

Figure S12. Different models predicting k1: this work (calculated with adapted blood flow), and models from literature (Arnot & Gobas, 2004), (Lee et al., 2002) and (Chen & Kuo, 2018); models that did not consider a reduced bioavailable fraction were multiplied by $f_{unbound}$.

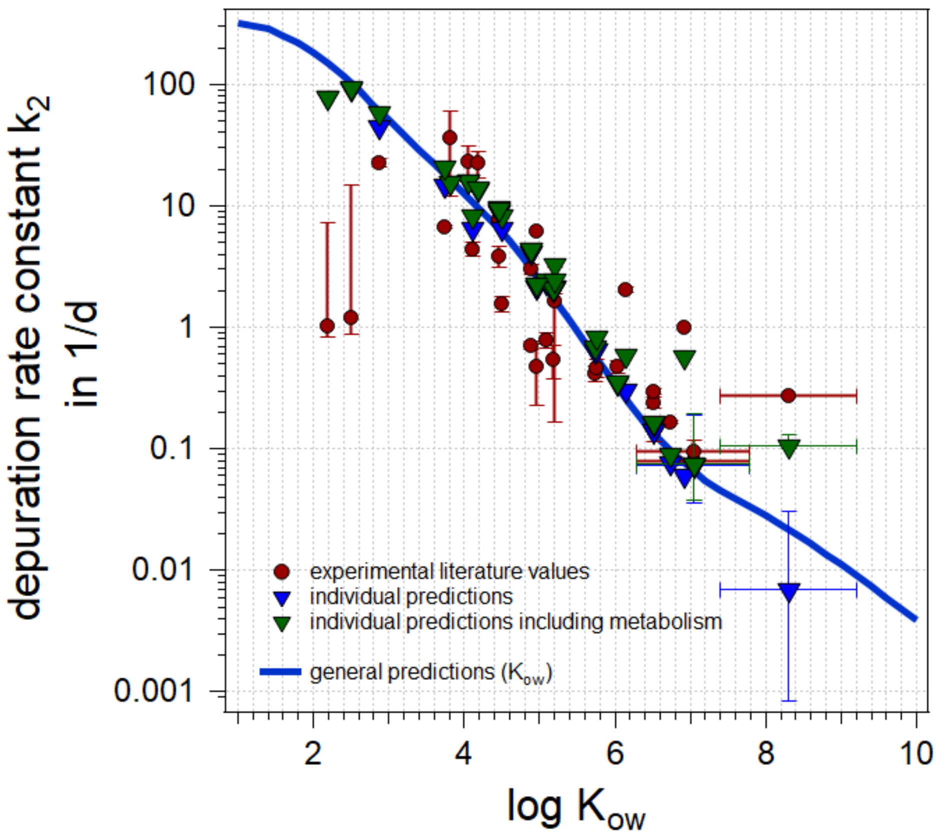

Figure S13. Predicted k2 according to Eq. (6), in the absence (blue) or presence (green) of metabolism, alongside experimental k2 (red) for blood flow modeled as in fish. Same chemicals taken from different literature are marked with a cross.

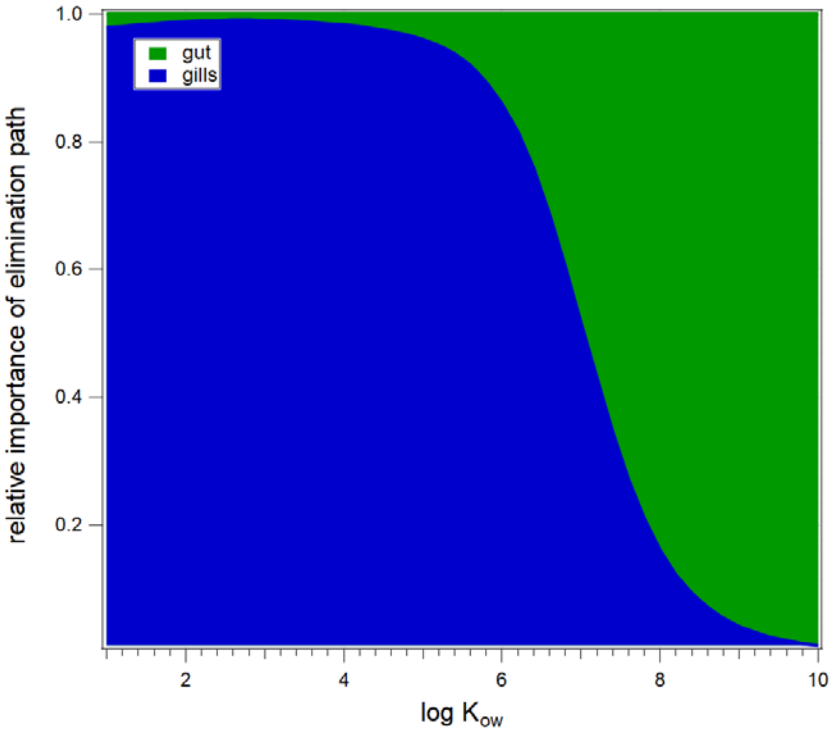

Figure S14. Relative importance of elimination paths in *H. azteca* in the absence of growth or metabolism. Blood flow is calculated with adapted blood flow.

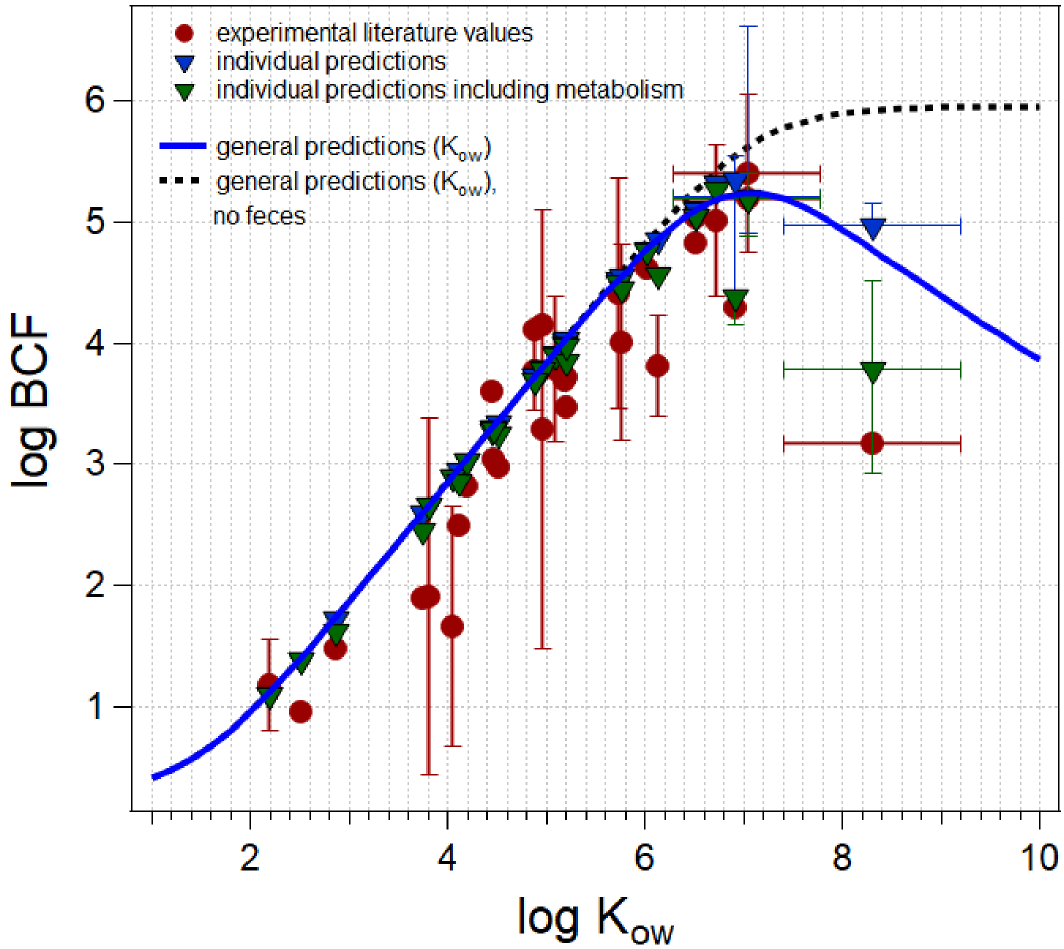

Figure S15. Predicted log BCF according to Eq.(7), in the absence (blue) or presence (green) of metabolism, alongside experimental k2 (red) for blood flow modeled as in fish. Same chemicals taken from different literature are marked with a cross.

Table S10: *Hyalella azteca*: Calculated uptake rate constant k1 for TOC content of 1 mg DOC/L, depuration rate constant k2, log BCF, and estimated times till 50% of steady state is reached, depending on the octanol/water partition coefficient log K_{ow}. Calculations were done with adapted blood flow.

Log K_{ow}	k1 ($L_w/kg_{org}/d$)	k2 (1/d)	Log BCF[a]	T50 (d)
1.0	1.94E+01	1.94E+01	0.40	0.036
1.2	2.21E+01	1.90E+01	0.46	0.037
1.4	2.57E+01	1.80E+01	0.55	0.039
1.6	3.08E+01	1.67E+01	0.66	0.042
1.8	3.78E+01	1.51E+01	0.80	0.046
2.0	4.74E+01	1.33E+01	0.95	0.052
2.2	6.06E+01	1.16E+01	1.12	0.060
2.4	7.86E+01	1.00E+01	1.29	0.069

Log K_{ow}	k1 ($L_w/kg_{org}/d$)	k2 (1/d)	Log BCF[a]	T50 (d)
2.6	1.03E+02	8.59E+00	1.48	0.081
2.8	1.37E+02	7.34E+00	1.67	0.094
3.0	1.82E+02	6.26E+00	1.86	0.11
3.2	2.43E+02	5.33E+00	2.06	0.13
3.4	3.26E+02	4.54E+00	2.25	0.15
3.6	4.37E+02	3.86E+00	2.45	0.18
3.8	5.87E+02	3.29E+00	2.65	0.21
4.0	7.87E+02	2.79E+00	2.85	0.25
4.2	1.05E+03	2.36E+00	3.05	0.29
4.4	1.39E+03	1.98E+00	3.24	0.35
4.6	1.82E+03	1.64E+00	3.44	0.42
4.8	2.33E+03	1.34E+00	3.64	0.52
5.0	2.93E+03	1.07E+00	3.83	0.65
5.2	3.58E+03	8.41E-01	4.03	0.82
5.4	4.26E+03	6.44E-01	4.22	1.08
5.6	4.90E+03	4.82E-01	4.4	1.44
5.8	5.45E+03	3.55E-01	4.58	1.95
6.0	5.87E+03	2.57E-01	4.76	2.7
6.2	6.08E+03	1.85E-01	4.91	3.74
6.4	6.06E+03	1.34E-01	5.05	5.19
6.6	5.78E+03	9.74E-02	5.17	7.12
6.8	5.25E+03	7.22E-02	5.26	9.6
7.0	4.50E+03	5.47E-02	5.31	12.7
7.2	3.64E+03	4.25E-02	5.33	16.3
7.4	2.79E+03	3.38E-02	5.31	20.5
7.6	2.03E+03	2.74E-02	5.27	25.3
7.8	1.41E+03	2.26E-02	5.19	30.7
8.0	9.57E+02	1.87E-02	5.11	37
8.2	6.33E+02	1.56E-02	5.01	44.3
8.4	4.13E+02	1.31E-02	4.9	53
8.6	2.66E+02	1.09E-02	4.79	63.5

Log K_{ow}	k1 ($L_w/kg_{org}/d$)	k2 (1/d)	Log BCF[a]	T50 (d)
8.8	1.71E+02	9.10E-03	4.67	76.2
9.0	1.09E+02	7.57E-03	4.56	91.6
9.2	6.93E+01	6.28E-03	4.44	110
9.4	4.40E+01	5.20E-03	4.32	133
9.6	2.79E+01	4.29E-03	4.21	161
9.8	1.76E+01	3.54E-03	4.1	196
10	1.12E+01	2.91E-03	3.98	239

[a] Predictions were done for *H. azteca* of 3 mg weight and 2% body fat content, BCF was normalized to 5% body fat content.

Table S11 Fish: Modeled uptake rate constants k1 for TOC content of 1 mg DOC/L, the log. BCF, k2 and estimated times till 50 % of steady state is reached, depending on the octanol/water partition coefficient log K_{ow}.

Log K_{ow}	k1 ($L_w/kg_{org}/d$)	k2 (1/d)	Log BCF[a]	T50 (d)
1.0	7.77E+01	6.33E+01	0.40	0.011
1.2	8.60E+01	5.83E+01	0.46	0.012
1.4	9.68E+01	5.17E+01	0.55	0.013
1.6	1.10E+02	4.42E+01	0.22	0.016
1.8	1.27E+02	3.64E+01	0.3	0.019
2.0	1.48E+02	2.91E+01	0.40	0.024
2.2	1.71E+02	2.27E+01	0.53	0.031
2.4	1.99E+02	1.72E+01	0.67	0.040
2.6	2.29E+02	1.29E+01	0.84	0.054
2.8	2.63E+02	9.48E+00	1.01	0.073
3.0	3.01E+02	6.93E+00	1.19	0.1
3.2	3.46E+02	5.05E+00	1.38	0.14
3.4	3.99E+02	3.70E+00	1.57	0.19
3.6	4.63E+02	2.72E+00	1.77	0.26
3.8	5.38E+02	2.00E+00	1.97	0.35
4.0	6.22E+02	1.46E+00	2.16	0.48
4.2	7.11E+02	1.06E+00	2.36	0.66

Log K_{ow}	k1 (L_w/kg_{org}/d)	k2 (1/d)	Log BCF[a]	T50 (d)
4.4	7.97E+02	7.49E-01	2.56	0.93
4.6	8.75E+02	5.21E-01	2.76	1.33
4.8	9.40E+02	3.55E-01	2.96	1.95
5.0	9.89E+02	2.39E-01	3.16	2.91
5.2	1.02E+03	1.58E-01	3.36	4.38
5.4	1.05E+03	1.04E-01	3.55	6.64
5.6	1.06E+03	6.87E-02	3.75	10.1
5.8	1.05E+03	4.55E-02	3.94	15.2
6.0	1.03E+03	3.05E-02	4.13	22.7
6.2	9.95E+02	2.08E-02	4.32	33.3
6.4	9.38E+02	1.45E-02	4.49	47.7
6.6	8.57E+02	1.04E-02	4.66	66.4
6.8	7.53E+02	7.73E-03	4.81	89.7
7.0	6.30E+02	5.90E-03	4.94	117
7.2	5.01E+02	4.62E-03	5.05	150
7.4	3.78E+02	3.68E-03	5.12	188
7.6	2.72E+02	2.97E-03	5.16	234
7.8	1.89E+02	2.40E-03	5.17	289
8.0	1.27E+02	1.94E-03	5.14	357
8.2	8.36E+01	1.56E-03	5.09	444
8.4	5.43E+01	1.25E-03	5.03	555
8.6	3.50E+01	9.95E-04	4.95	697
8.8	2.23E+01	7.91E-04	4.86	876
9.0	1.42E+01	6.28E-04	4.77	1100
9.2	9.03E+00	4.99E-04	4.68	1390
9.4	5.72E+00	3.96E-04	4.58	1750
9.6	3.62E+00	3.15E-04	4.49	2200
9.8	2.29E+00	2.50E-04	4.39	2770
10	1.45E+00	1.99E-04	4.29	3490

[a] Calculated with 3.7 % lipid content in 2.2g fish, and normalized to 5%.

Supporting References

Arnot, J. A., & Gobas, F. A. P. C. (2004). A food web bioaccumulation model for organic chemicals in aquatic ecosystems. *Environmental Toxicology and Chemistry*, *23*(10), 2343–2355. https://doi.org/10.1897/03-438

Avdeef, A. (2010). Leakiness and Size Exclusion of Paracellular Channels in Cultured Epithelial Cell Monolayers–Interlaboratory Comparison. *Pharmaceutical Research*, *27*(3), 480–489. https://doi.org/10.1007/s11095-009-0036-7

Baker, E. A., Hayes, A. L., & Butler, R. C. (1992). Physicochemical properties of agrochemicals: Their effects on foliar penetration. *Pesticide Science*, *34*(2), 167–182. https://doi.org/10.1002/ps.2780340212

Benson, B. B., & Krause, D. (1980). The concentration and isotopic fractionation of gases dissolved in freshwater in equilibrium with the atmosphere. 1. Oxygen. *Limnology and Oceanography*, *25*(4), 662–671. https://doi.org/10.4319/lo.1980.25.4.0662

Bittermann, K., & Goss, K. U. (2017). Predicting apparent passive permeability of Caco-2 and MDCK cell-monolayers: A mechanistic model. *PLoS ONE*, *12*(12), 1–20. https://doi.org/10.1371/journal.pone.0190319

Brown, T. N., Arnot, J. A., & Wania, F. (2012). Iterative fragment selection: A group contribution approach to predicting fish biotransformation half-lives. *Environmental Science and Technology*, *46*(15), 8253–8260. https://doi.org/10.1021/es301182a

Buddington, R. K., & Diamond, J. M. (1987). Pyloric ceca of fish: A “new” absorptive organ. *American Journal of Physiology - Gastrointestinal and Liver Physiology*, *252*(1 (15/1)). https://doi.org/10.1152/ajpgi.1987.252.1.g65

Chen, C. C., & Kuo, D. T. F. (2018). Bioconcentration model for non-ionic, polar, and ionizable organic compounds in amphipod. *Environmental Toxicology and Chemistry*, *37*(5), 1378–1386. https://doi.org/10.1002/etc.4081

De Maagd, P. G. J., Ten Hulscher, D. T. H. E. M., Van Den Heuvel, H., Opperhuizen, A., & Sijm, D. T. H. M. (1998). Physicochemical properties of polycyclic aromatic hydrocarbons: Aqueous solubilities, n-octanol/water partition coefficients, and Henry’s law constants. *Environmental Toxicology and Chemistry*, *17*(2), 251–257. https://doi.org/10.1897/1551-5028(1998)017<0251:PPOPAH>2.3.CO;2

Do, A. T. N., Kim, Y., Ha, Y., & Kwon, J. H. (2022). Estimating the Bioaccumulation Potential of Hydrophobic Ultraviolet Stabilizers Using Experimental Partitioning Properties. *International Journal of Environmental Research and Public Health*, *19*(7). https://doi.org/10.3390/ijerph19073989

EAS-E Suite. (2022). *(Ver.0.95 - BETA; release Feb.; 2022).* www.eas-e-suite.com. Developed by ARC Arnot Research and Consulting Inc., Toronto, ON, Canada

Eckert, F., & Klamt, A. (2002). Fast solvent screening via quantum chemistry: COSMO-RS approach. *AIChE Journal*, *48*(2), 369–385. https://doi.org/10.1002/aic.690480220

Endo, S., & Goss, K. U. (2011). Serum albumin binding of structurally diverse neutral organic compounds: Data and models. *Chemical Research in Toxicology*, *24*(12), 2293–2301. https://doi.org/10.1021/tx200431b

Erickson, R. J., & McKim, J. M. (1990). A model for exchange of organic chemicals at fish gills: flow and diffusion limitations. *Aquatic Toxicology*, *18*(4), 175–197. https://doi.org/10.1016/0166-445X(90)90001-6

Escher, B. I., Cowan-Ellsberry, C. E., Dyer, S., Embry, M. R., Erhardt, S., Halder, M., Kwon, J. H., Johanning, K., Oosterwijk, M. T. T., Rutishauser, S., Segner, H., & Nichols, J. (2011). Protein and lipid binding parameters in rainbow trout (Oncorhynchus mykiss) blood and liver fractions to extrapolate from an in vitro metabolic degradation assay to in vivo bioaccumulation potential of hydrophobic organic chemicals. *Chemical Research in Toxicology*, *24*(7), 1134–1143. https://doi.org/10.1021/tx200114y

Everitt, S., MacPherson, S., Brinkmann, M., Wiseman, S., & Pyle, G. (2020). Effects of weathered sediment-bound dilbit on freshwater amphipods (Hyalella azteca). *Aquatic Toxicology*, *228*(June), 105630. https://doi.org/10.1016/j.aquatox.2020.105630

Fitzpatrick, C. M. (1968). *The population dynamics and bioenergetics of the isopod Asellus aquaticus L. in a small freshwater pond*. Durham University.

Gaigalas, A. K., Hubbard, J. B., McCurley, M., & Woo, S. (1992). Diffusion of bovine serum albumin in aqueous solutions. *Journal of Physical Chemistry*, *96*(5), 2355–2359. https://doi.org/10.1021/j100184a063

Gauthier, P. T., Norwood, W. P., Prepas, E. E., & Pyle, G. G. (2016). Behavioural alterations from exposure to Cu, phenanthrene, and Cu-phenanthrene mixtures: Linking behaviour to acute toxic mechanisms in the aquatic amphipod, Hyalella azteca. *Aquatic Toxicology*, *170*, 377–383. https://doi.org/10.1016/j.aquatox.2015.10.019

Gingerich, W. H., Pityer, R. A., & Rach, J. J. (1987). Estimates of plasma, packed cell and total blood volume in tissues of the rainbow trout (Salmo gairdneri). *Comparative Biochemistry and Physiology -- Part A: Physiology*, *87*(2), 251–256. https://doi.org/10.1016/0300-9629(87)90119-8

Halcrow, K. (2001). Ultrastructural features of the funnel of Gammarus oceanicus (Amphipoda). *Journal of Crustacean Biology*, *21*(3), 631–639. https://doi.org/10.1163/20021975-99990164

Hansch, C., Leo, A., & Hoekman, D. H. (1995). *Exploring QSAR.: Hydrophobic, electronic, and steric constants*. American Chemical Society.

Johanif, N., Huff Hartz, K. E., Figueroa, A. E., Weston, D. P., Lee, D., Lydy, M. J., Connon, R. E., & Poynton, H. C. (2021). Bioaccumulation potential of chlorpyrifos in resistant Hyalella azteca: Implications for evolutionary toxicology. *Environmental Pollution*, *289*(July), 117900. https://doi.org/10.1016/j.envpol.2021.117900

Johnke, R. (1973). The influence of season upon the oxygen consumption of two populations of the freshwater amphipod hyalella azteca. *Master Thesis (Ocm60457213), California State University, Fresno*, *August*. http://hdl.handle.net/20.500.12680/nz806233d

Kampfraath, A. A., Hunting, E. R., Mulder, C., Breure, A. M., Gessner, M. O., Kraak, M. H. S., & Admiraal, W. (2012). DECOTAB: A multipurpose standard substrate to assess effects of litter quality on microbial decomposition and invertebrate consumption. *Freshwater Science*, *31*(4), 1156–1162. https://doi.org/10.1899/12-075.1

Karickhoff, S. W., Brown, D. S., & Scott, T. A. (1979). Sorption of hydrophobic pollutants on natural sediments. *Water Research*, *13*(3), 241–248. https://doi.org/10.1016/0043-1354(79)90201-X

Kosfeld, V., Fu, Q., Ebersbach, I., Esser, D., Schauerte, A., Bischof, I., Hollender, J., & Schlechtriem, C. (2020). Comparison of Alternative Methods for Bioaccumulation Assessment: Scope and Limitations of In Vitro Depletion Assays with Rainbow Trout and Bioconcentration Tests in the Freshwater Amphipod Hyalella azteca. *Environmental Toxicology and Chemistry*, *39*(9), 1813–1825. https://doi.org/10.1002/etc.4791

Krause, S., Ulrich, N., & Goss, K. U. (2018). Desorption kinetics of organic chemicals from albumin. *Archives of Toxicology*, *92*(3), 1065–1074. https://doi.org/10.1007/s00204-017-2117-4

Landrum, P. F., & Scavia, D. (1983). Influence of sediment on anthracene uptake, depuration, and biotransformation by the amphipod Hyalella azteca. *Canadian Journal of Fisheries and Aquatic Sciences*, *40*(3), 298–305. https://doi.org/10.1139/f83-044

Landrum, P. F., Steevens, J. A., Gossiaux, D. C., McElroy, M., Robinson, S., Begnoche, L., Chernyak, S., & Hickey, J. (2004). Time-dependent lethal body residues for the toxicity of pentachlorobenzene to Hyalella azteca. *Environmental Toxicology and Chemistry*, *23*(5), 1335–1343. https://doi.org/10.1897/03-164

Landrum, P. F., Steevens, J. A., McElroy, M., Gossiaux, D. C., Lewis, J. S., & Robinson, S. D. (2005). Time-dependent toxicity of dichlorodiphenyldichloroethylene to Hyalella azteca. *Environmental Toxicology and Chemistry*, *24*(1), 211–218. https://doi.org/10.1897/04-055R.1

Larisch, W. (2019). Entwicklung eines PbTk-Modells für Wirbeltiere. *Dissertation, Martin-Luther-Universität Halle-Wittenberg*.

Larisch, W., Brown, T. N., & Goss, K. U. (2017). A toxicokinetic model for fish including multiphase sorption features. *Environmental Toxicology and Chemistry*, *36*(6), 1538–1546. https://doi.org/10.1002/etc.3677

Larisch, W., & Goss, K. U. (2018a). Modelling oral up-take of hydrophobic and super-hydrophobic chemicals in fish. *Environmental Science: Processes and Impacts*, *20*(1), 98–104. https://doi.org/10.1039/c7em00495h

Larisch, W., & Goss, K. U. (2018b). Modelling oral up-take of hydrophobic and super-hydrophobic chemicals in fish. *Environmental Science: Processes and Impacts*, *20*(1), 98–104. https://doi.org/10.1039/c7em00495h

Lee, J. H., Landrum, P. F., & Koh, C. H. (2002). Toxicokinetics and time-dependent PAH toxicity in the amphipod Hyalella azteca. *Environmental Science and Technology*, *36*(14), 3124–3130. https://doi.org/10.1021/es011201l

Liu, L., Wu, F., Haderlein, S., & Grathwohl, P. (2013). Determination of the subcooled liquid solubilities of PAHs in partitioning batch experiments. *Geoscience Frontiers*, *4*(1), 123–126. https://doi.org/10.1016/j.gsf.2012.03.010

Lotufo, G. R., Landrum, P. F., Gedeon, M. L., Tigue, E. A., & Herche, L. R. (2000). Comparative toxicity and toxicokinetics of DDT and its major metabolites in freshwater amphipods. *Environmental Toxicology and Chemistry*, *19*(2), 368–379. https://doi.org/10.1002/etc.5620190217

Mansouri, K., Grulke, C. M., Richard, A. M., Judson, R. S., & Williams, A. J. (2016). An automated curation procedure for addressing chemical errors and inconsistencies in public datasets used in QSAR modelling. *SAR and QSAR in Environmental Research*, *27*(11), 939–965. https://doi.org/10.1080/1062936X.2016.1253611

Morgan, M. (1971). *Gill development, growth and respiration in the trout, Salmo gairdneri (Richardson)*. Bristol University.

Naeem, M., Salam, A., & Zuberi, A. (2016). Proximate composition of freshwater rainbow trout (Oncorhynchus mykiss) in relation to body size and condition factor from Pakistan. *Pakistan Journal of Agricultural Sciences*, *53*(2), 468–472. https://doi.org/10.21162/PAKJAS/16.2653

Nichols, J. W., McKim, J. M., Lien, G. J., Hoffman, A. D., Bertelsen, S. L., & Elonen, C. M. (1996). A physiologically based toxicokinetic model for dermal absorption of organic chemicals by fish. *Fundamental and Applied Toxicology*, *31*(2), 229–242. https://doi.org/10.1006/faat.1996.0095

Nuutinen, S., Landrum, P. F., Schuler, L. J., Kukkonen, J. V. K., & Lydy, M. J. (2003). Toxicokinetics of organic contaminants in Hyalella azteca. *Archives of Environmental Contamination and Toxicology*, *44*(4), 467–475. https://doi.org/10.1007/s00244-002-2127-x

Othman, M. S., & Pascoe, D. (2001). Growth, development and reproduction of Hyalella Azteca (Saussure, 1858) in laboratory culture. *Crustaceana*, *74*(2), 171–181. https://doi.org/10.1163/156854001750096274

Schlechtriem, C., Kampe, S., Bruckert, H. J., Bischof, I., Ebersbach, I., Kosfeld, V., Kotthoff, M., Schäfers, C., & L'Haridon, J. (2019). Bioconcentration studies with the freshwater amphipod Hyalella azteca: are the results predictive of bioconcentration in fish? *Environmental Science and Pollution Research*, *26*(2), 1628–1641. https://doi.org/10.1007/s11356-018-3677-4

Schlechtriem, C., Kühr, S., & Müller, C. (2022). *Development of a bioaccumulation test using Hyalella azteca*.

Schuler, L. J., Landrum, P. F., & Lydy, M. J. (2004). Time-dependent toxicity of fluoranthene to freshwater invertebrates and the role of biotransformation on lethal body residues. *Environmental Science and Technology*, *38*(23), 6247–6255. https://doi.org/10.1021/es049844z

Sushko, I., Novotarskyi, S., Körner, R., Pandey, A. K., Rupp, M., Teetz, W., Brandmaier, S., Abdelaziz, A., Prokopenko, V. V., Tanchuk, V. Y., Todeschini, R., Varnek, A., Marcou, G., Ertl, P., Potemkin, V., Grishina, M., Gasteiger, J., Schwab, C., Baskin, I. I., … Tetko, I. V. (2011). Online chemical modeling environment (OCHEM): Web platform for data storage, model development and publishing of chemical information. *Journal of Computer-Aided Molecular Design*, *25*(6), 533–554. https://doi.org/10.1007/s10822-011-9440-2

SUTCLIFFE, D. W. (1984). Quantitative aspects of oxygen uptake by Gammarus (Crustacea, Amphipoda): a critical review. *Freshwater Biology*, *14*(5), 443–489. https://doi.org/10.1111/j.1365-2427.1984.tb00168.x

Tomlin, C. D. S. (2003). *The Pesticide Manual.* (13th Editi). British Crop Protection Council, UK.

Ulrich, N., Endo, S., Brown, T. N., Watanabe, N., Bronner, G., Abraham, M. H., & Goss, K. U. (2017). *UFZ-LSER database v 3.2 [Internet]*. http://www.ufz.de/lserd

Verkman, A. S. (2002). Solute and macromolecule diffusion in cellular aqueous compartments. *Trends in Biochemical Sciences*, *27*(1), 27–33. https://doi.org/10.1016/S0968-0004(01)02003-5

Walter, A., & Gutknecht, J. (1986). Permeability of small nonelectrolytes through lipid bilayer membranes. *The Journal of Membrane Biology*, *90*(3), 207–217. https://doi.org/10.1007/BF01870127

Welton, J. S., Ladle, M., Bass, J. A. B., & John, I. R. (1983). Estimation of Gut Throughput Time in Gammarus Pulex under Laboratory and Field Conditions with a Note on the Feeding of Young in the Brood Pouch. *Oikos*, *41*(1), 133. https://doi.org/10.2307/3544355

Westergaard, H., & Dietschy, J. M. (1976). The mechanism whereby bile acid micelles increase the rate of fatty acid and cholesterol uptake into the intestinal mucosal cell. *Journal of Clinical Investigation*, *58*(1), 97–108. https://doi.org/10.1172/JCI108465
